# Cobamide metabolism, regulation, and adaptation in *Mycobacterium tuberculosis*

**DOI:** 10.1128/jb.00204-25

**Published:** 2025-11-11

**Authors:** Terry Kipkorir, Rendani D. Mbau, Digby F. Warner, Gopinath Krishnamoorthy, Atica Moosa

**Affiliations:** 1 Department of Infection Biology, The London School of Hygiene and Tropical Medicine 4906, London, United Kingdom; 2 SAMRC Centre for Tuberculosis Research, Division of Molecular Biology and Human Genetics, Department of Biomedical Sciences, Faculty of Medicine and Health Sciences, Stellenbosch University 26697, Cape Town, South Africa; 3 Molecular Mycobacteriology Research Unit, Division of Medical Microbiology, Department of Pathology, University of Cape Town 37716, Cape Town, South Africa; 4 Institute of Infectious Disease and Molecular Medicine, University of Cape Town 37716, Cape Town, South Africa; 5 Wellcome Discovery Research Platform for Infection, CIDRI-Africa, University of Cape Town 37716, Cape Town, South Africa; 6 Department of Infectious Diseases, Respiratory Medicine and Critical Care, Charité – Universitätsmedizin Berlin, Corporate Member of Freie Universität Berlin, Humboldt-Universität zu Berlin 9373, Berlin, Germany; Geisel School of Medicine at Dartmouth, Hanover, New Hampshire, USA

**Keywords:** corrinoids, methionine synthase, ribonucleotide reductase, methylmalonyl-CoA mutase, B_12_ riboswitch, Vitamin B_12_

## Abstract

Cobamides play a paradoxical but critical role in the biology of *Mycobacterium tuberculosis* (*Mtb*), the causative agent of tuberculosis. Although *Mtb* retains nearly all cobalamin (Cbl) biosynthetic genes and encodes multiple cobamide-requiring enzymes, experimental evidence indicates that *Mtb* is incapable of *de novo* Cbl synthesis under any tested conditions to date. Instead, an evolutionary shift appears to have occurred toward host dependency for biologically relevant cobamides or their precursors. This review highlights recent advances in our understanding of cobamide-related metabolism in *Mtb*, including: (i) the progressive erosion of *de novo* cobamide biosynthetic capacity across *Mtb* lineages; (ii) the role of host-derived cobamides in sustaining key mycobacterial metabolic pathways, including methionine synthesis and propionate catabolism; (iii) the impact of host immune pressures, including itaconate-mediated inhibition of methylmalonyl-CoA mutase; (iv) strategies employed by *Mtb* for cobamide and precursor acquisition; and (v) unique adaptations of Cbl-sensing riboswitches that regulate methionine synthesis, virulence-associated gene expression, and dormancy resuscitation. We also highlight unresolved questions, including possible niche-specific synthesis, utilization of alternate cobamide species, and the therapeutic potential of targeting cobamide-related metabolism. We review recent evidence of the centrality of cobamides in the metabolic flexibility of *Mtb*, virulence, and survival in the host environment, despite apparent loss of *de novo* biosynthetic capacity. Further mechanistic studies are required which may reveal vulnerabilities for the exploitation of cobamide acquisition, cobamide-related regulation, and the role of cobamides at the *Mtb*-host interface for innovative therapeutic interventions.

## A FOREWORD ON NOMENCLATURE

**Fig 1:**
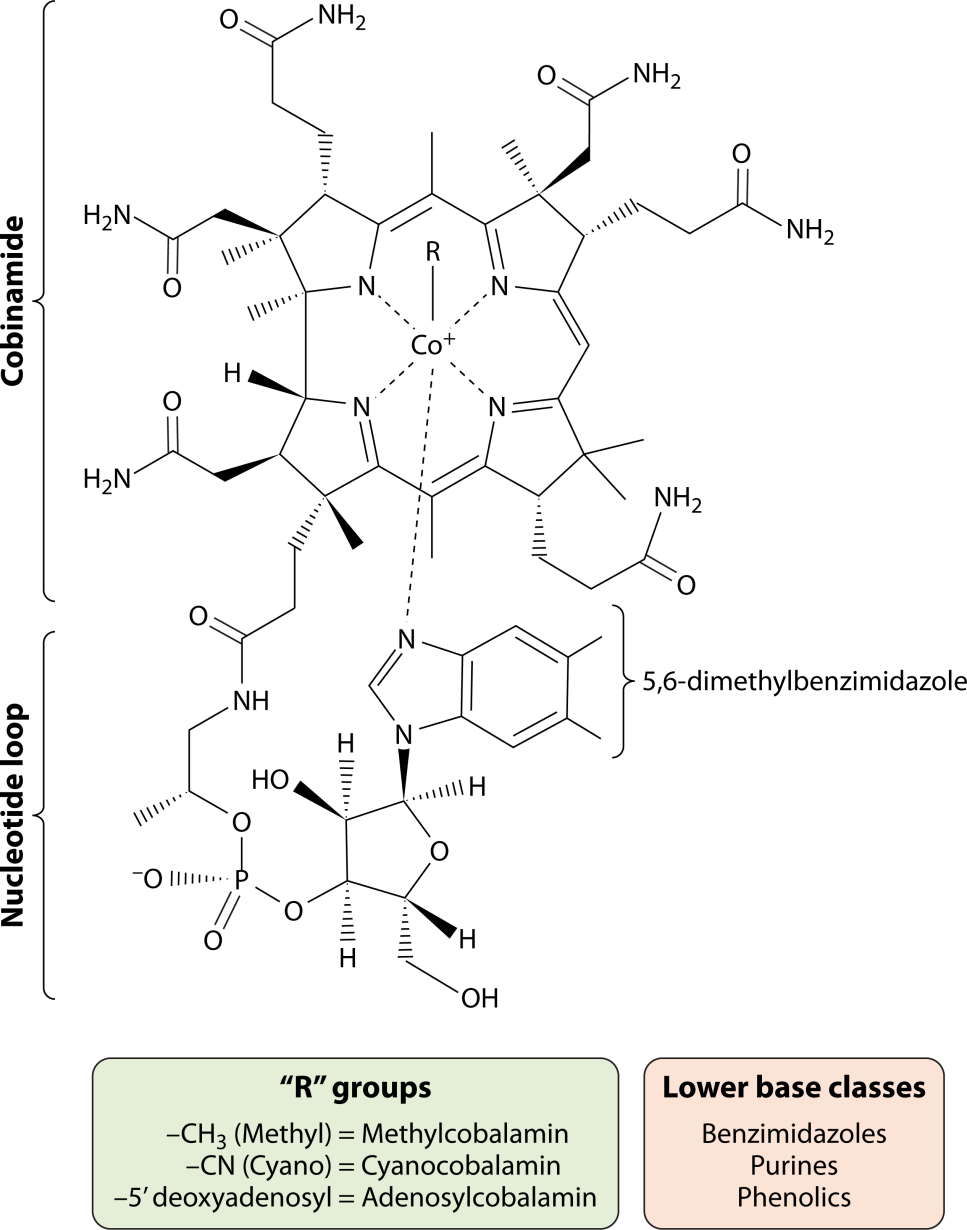
Chemical structure and diversity of cobamides. Cobamides consist of a corrin ring with a centrally chelated cobalt ion and a nucleotide loop that includes a lower ligand base. In Cbl, the lower base is DMB, but other cobamides may incorporate purines or phenolic compounds as alternative lower bases. The upper axial ligand (R group) is also variable and defines the specific cobamide isoform. Cobinamide refers to the incomplete form of a cobamide that lacks the nucleotide loop and lower base.

Cobamides are among the largest and most structurally complex cofactors in nature. However, terminology surrounding this class of compounds has been inconsistent (1). For consistency, we define cobamides as molecules consisting of a cobalt-chelated corrin ring linked to a lower nucleotide loop, with structural diversity arising from variation in the base of this loop (Fig. 1) (2–4). The cobalt ion is coordinated at the α-axial position by a lower base attached to the corrin ring via an aminopropanol linker, forming the nucleotide loop, while a variable upper ligand (a.k.a “R” group) coordinates the cobalt at the β-axial position (Fig. 1) (2–4). We distinguish between cobalamin (Cbl), characterized by its 5,6-dimethylbenzimidazole (DMB) lower ligand, and vitamin B_12_ or cyanocobalamin (CNCbl), the commercially produced form of Cbl, widely used in clinical and nutritional contexts, but is inactive in cobalamin-dependent enzymes. Comparatively, adenosylcobalamin (AdoCbl) functions as the coenzyme form of Cbl in radical-mediated enzymatic reactions, while methylcobalamin (MeCbl) represents the cofactor form and serves as a methyl donor in methyltransferase reactions. For MeCbl, the catalytically active species is the reduced Co(I)cobamide, which accepts the methyl group during turnover; the subsequent methyl transfer occurs from the Me-Co(III) state (5, 6). We also note the existence of Cbl analogs, which differ in their lower ligand composition, displaying benzimidazoles, purines, or phenolic bases, but retain cobalt and the corrin ring (Fig. 1). Here, we use the term “incomplete corrinoids” to refer to compounds such as cobinamide or cobyric acid that retain the corrin but lack the aminopropanol linker and lower base. Widely in the literature, including our previous publications, “B_12_-dependent” is a term used to describe cobamide-requiring enzymes, often in contexts that do not identify the precise upper ligand or redox state. In this review, we favor terminology that reflects both structural and mechanistic distinctions and refer to these enzymes as Cbl-dependent, specifying AdoCbl- or MeCbl-dependent where known. Because supplementation with CNCbl supports growth *in vitro* (as discussed in later sections)*,* these enzymes are presumably functional when the organism is fed CNCbl, suggesting that *Mycobacterium tuberculosis* (*Mtb*) can remodel cyanocobamides under certain conditions.

## BIOSYNTHESIS OF COBAMIDES IN MYCOBACTERIA

Cobamides are essential cofactors in diverse metabolic processes across all domains of life, yet their *de novo* biosynthesis is limited to a narrow group of bacterial and archaeal species (2, 3, 7–9). Most organisms, including humans and animals, must obtain the relevant cobamide through their diet or symbiotic microbial interactions. In mammals, cobamides are required for the function of two enzymes: methionine synthase (MetH), located in the cytosol, and methylmalonyl-CoA mutase (MCM), which is mitochondrion associated (10, 11). Because of their biochemical and clinical importance, cobamide deficiency can cause severe physiological consequences, including anemia, neurological impairments, and developmental defects (12).

**Fig 2:**
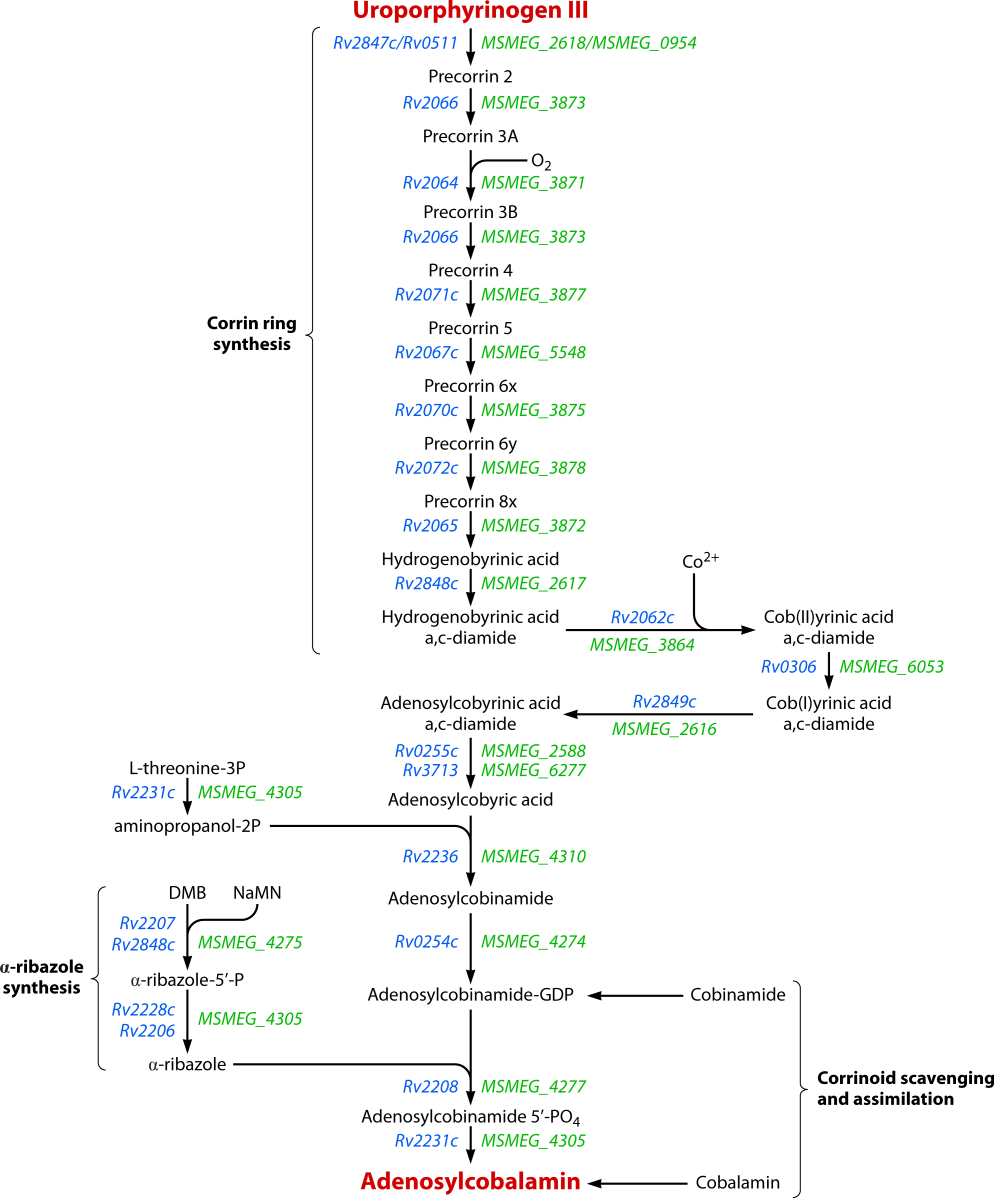
Predicted aerobic pathway for AdoCbl biosynthesis in mycobacteria. A reconstruction of the canonical *de novo* biosynthetic pathway for AdoCbl from uroporphyrinogen III. Gene annotations are shown for both *Mtb* H37Rv (Rv numbers) and *Mycobacterium smegmatis* MC²155 (MSMEG numbers), as curated in the Mycobrowser database (https://mycobrowser.epfl.ch/; [19]). While *M. smegmatis* retains a complete and functional pathway, *Mtb* is thought to have lost the capacity for *de novo* cobamide synthesis. Instead, *Mtb* likely relies on salvage and conversion of cobinamide and related precursors to AdoCbl. The schematic also includes the parallel biosynthetic route for α-ribazole, which combines with adenosylcobinamide to form the complete cobamide.

In cobamide producers, biosynthesis is remarkably complex, requiring approximately 30 enzymatic steps, proceeding from the common intermediate, uroporphyrinogen III, requiring insertion and coordination of the cobalt ion within the corrin ring, and finally, assembly of the nucleotide loop during late steps of the pathway (Fig. 2) (13). Genomic analyses have revealed that *Mtb* harbors a nearly complete set of genes for *de novo* Cbl biosynthesis (Fig. 2), along with three putative cobamide-requiring enzymes (Table 1) (14, 15). Yet, despite the extensive genomic commitment to cobamide-related metabolism, no evidence generated under controlled experimental conditions indicates that *Mtb* can synthesize Cbl either *in vitro* or *in vivo* (16–18). The disconnect between the inferred genetic potential and demonstrated metabolic output raises fundamental questions about evolutionary forces shaping cobamide-related metabolism in *Mtb*.

**TABLE 1:** Cobamide-requiring enzymes in *Mtb^a^*

Enzyme and reaction catalyzed	Gene name/(locus)	Note
$\begin{array}{l} Methylmalonyl-CoA\;mutase\\ (R)\text{-}2\text{-}methyl\text{-}malonyl\text{-}CoA\overset{}{\underset{AdoCbl}{\to }}Succinyl\text{-}CoA \end{array}$	*mutAB* (*Rv1492*/*Rv1493*)	MutAB has an anaplerotic role and acts as a bypass to AdoCbl-independent methylcitrate and glyoxylate cycles in propionate catabolism (20–23)
$\begin{array}{l} Methionine\;synthase\\ 5\text{-}methyltetrahydrofolate+L\text{-}homocysteine\overset{}{\underset{MeCbl}{\to }}tetrahydrofolate+L\text{-}methionine \end{array}$	*metH* (*Rv2124c*)	Disruption of *metE* causes cobamide auxotrophy; *metH* deletion leads to cobamide sensitivity in lab and clinical *Mtb* strains (16)
$\begin{array}{l} Ribonucleotide\;reductase\;(RNRs)\\ 2^{\prime }\text{-}deoxyribonucleoside\;diphosphate+Trx_{\left(ox\right)}+H_{2}O\;\overset{}{\underset{AdoCbl}{↔}}\;ribonucleoside\;diphosphate+Trx_{\left(red\right)} \end{array}$	*nrdZ* (*Rv0570*)	The class II RNR NrdZ requires AdoCbl and functions independently of oxygen, unlike class I (oxygen dependent) and class III (oxygen sensitive) RNRs. *Mtb* encodes multiple class I RNRs; *nrdEF2* (class Ib) is essential for *in vitro* growth. *nrdZ* is predicted but non-essential *in vitro* and in mouse infection (24, 25)

^*a*^ Trx_(ox)_, oxidized thioredoxin; Trx_(red)_, reduced thioredoxin.

Since the publication of our previous review on this subject (14), significant advances have deepened our understanding of how cobamide biosynthesis, transport, and utilization are regulated in *Mtb*, and how these pathways might intersect during host-pathogen interactions. Specifically, recent genomic, biochemical, and *in vivo* studies have shed light on strain-specific adaptations (17, 18, 26–28), the functional significance of retained cobamide-related genes (14, 16–18, 20, 26), and the impact of host immune pressures, such as itaconate production and Cbl limitation, on metabolic strategies employed by *Mtb* (18, 29–33).

In this update, we synthesize findings from the past decade to provide a comprehensive overview of cobamide-related metabolism in *Mtb* and other mycobacteria, focusing on: (i) the functional relevance of cobamide-requiring enzymes and Cbl-mediated regulatory mechanisms involving riboswitches and upstream open reading frames (uORFs) for *Mtb* pathogenesis; (ii) strategies for cobamide acquisition and exploitation of host-derived corrinoids; (iii) the evolutionary shift from cobamide production in an environmental progenitor to a host-dependent pathogenic lifestyle; (iv) metabolic adaptation of *Mtb* to host immune pressures; and (v) unresolved questions and future directions, including whether *Mtb* retains latent capacity for cobamide synthesis under untested conditions, outstanding structural biology questions surrounding key enzymes and riboswitches, and potential therapeutic opportunities for targeting cobamide-related pathways.

## COBAMIDE-REQUIRING ENZYMES IN MYCOBACTERIAL SPECIES

The *Mtb* genome encodes three enzymes predicted to require a cobamide for activity (Table 1; reviewed in reference 14). While AdoCbl is used by MCM and the class II ribonucleotide reductase (NrdZ), MeCbl functions as a cofactor for MetH in the conversion of homocysteine to methionine.

Class II ribonucleotide reductases are oxygen independent, in contrast to class I and class III, which are oxygen dependent or strictly anaerobic, respectively (24). AdoCbl-dependent NrdZ is dispensable for growth *in vitro* and in a mouse model. However, its participation in the mycobacterial DosRS dormancy response transcriptional network, a two-component regulatory system that mediates transcriptional adaptation to hypoxia and nitric oxide, facilitating entry into a non-replicating persistent state, suggests a role in hypoxic adaptation of *Mtb* (24).

**Fig 3:**
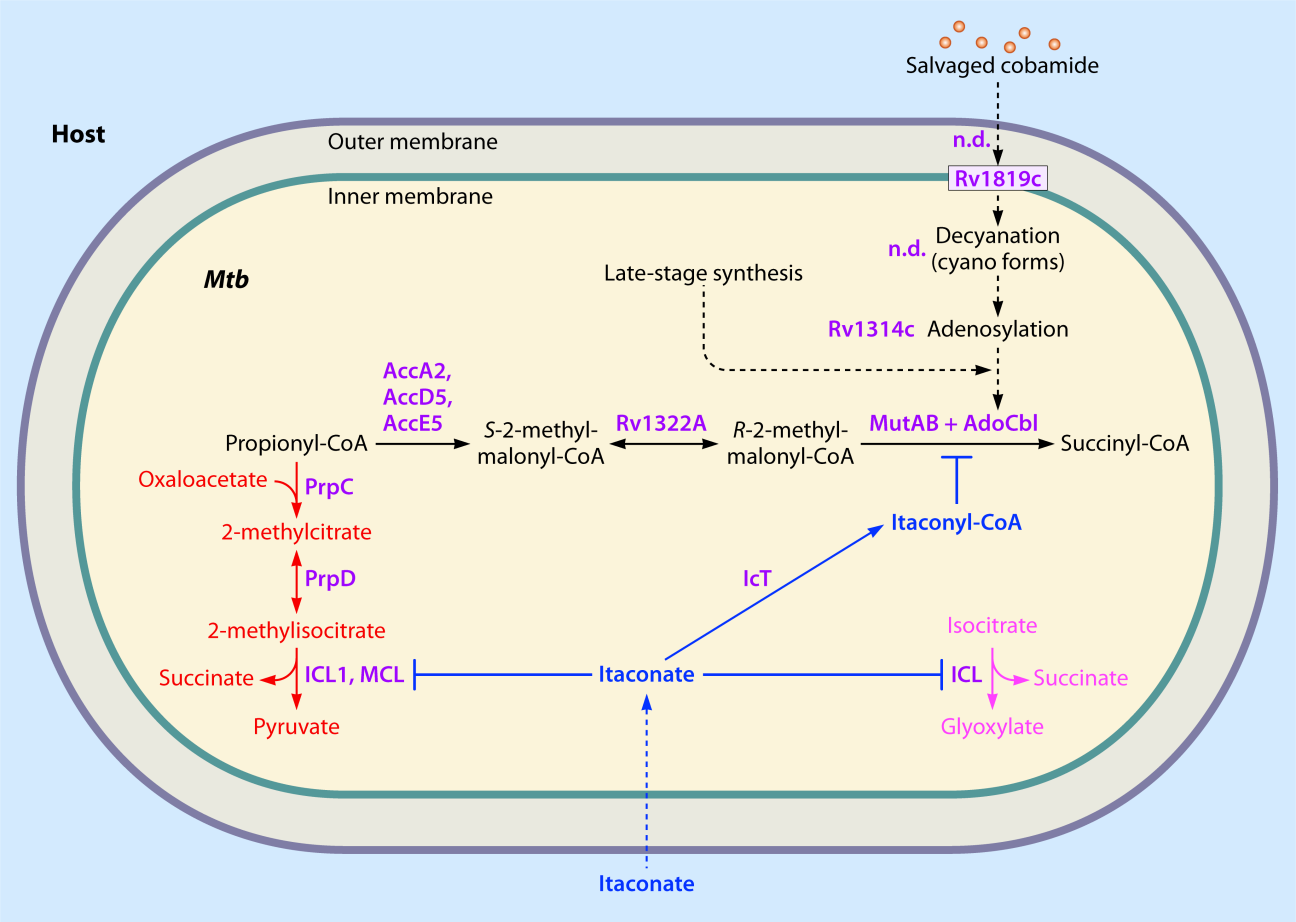
Itaconate-mediated disruption of methylmalonyl-CoA mutase activity in intracellular *Mtb.* This schematic illustrates proposed steps in the intracellular processing of cobinamide or cyanocobalamin (CNCbl) by *Mtb* and their intersection with host-derived itaconate stress. While the mechanism of cobamide transport across the mycomembrane remains unresolved (n.d.), the inner membrane ABC transporter Rv1819c is implicated in cytosolic import. Decyanation steps are also not determined (n.d.). Internalised incomplete cobamides are adenosylated by the PduO-type adenosyltransferase Rv1314c to form AdoCbl, the active cofactor for MCM, which detoxifies propionyl-CoA. Host-derived itaconate is converted to itaconyl-CoA, which irreversibly inactivates MCM by forming a stable adduct with AdoCbl. Itaconate also inhibits isocitrate lyase (ICL) and methylcitrate lyase (MCL), disrupting lipid metabolism and intracellular persistence.

The AdoCbl-dependent MCM is relatively well characterized in *Mtb*. This enzyme catalyzes the reversible isomerization of *R*-methylmalonyl-CoA to succinyl-CoA, the final step in the methylmalonyl pathway (Fig. 3). This reaction plays a critical role in modulating intracellular pools of propionyl-CoA, a potentially toxic byproduct arising from the catabolism of odd- and branched-chain fatty acids, branched-chain amino acids, and cholesterol (20, 21). Recently, it was shown that depletion of methylmalonyl-CoA inhibits the growth of *Mtb* strains capable of producing phthiocerol dimycocerosate (PDIM), a long-chain nonpolar lipid required for full virulence and maintenance of the cell membrane barrier (34). These results underscore the necessity of an optimal AdoCbl supply to support propionate assimilation and sustain PDIM biosynthesis in *Mtb*. The central role of PDIM in establishing infection in macrophages and murine models may underlie host immune effectors specifically targeting propionate metabolism as part of their antimycobacterial strategy (20, 22, 23, 29, 30, 35).

Immunomodulators such as itaconate and its derivatives regulate macrophage metabolism and inhibit key *Mtb* enzymes involved in the assimilation of propionyl-CoA (20, 22, 23, 29, 30). Notably, itaconyl-CoA, the coenzyme A derivative of itaconate, irreversibly inhibits MCM activity by forming a stable, non-repairable adduct with the AdoCbl cofactor (Fig. 3) (29, 30). This inactivation blocks MCM function, illustrating how host-derived metabolites can directly disrupt AdoCbl-dependent enzymatic pathways within *Mtb*. Together, these findings emphasize that AdoCbl availability modulates the dynamic interplay between host immune metabolism and pathogenic survival strategies through its influence on *Mtb* MCM activity.

In *Mtb*, two methionine synthases, MetH and MetE, catalyze the conversion of homocysteine to methionine, an essential amino acid necessary for one-carbon metabolism (16, 36). Like its human counterpart, *Mtb* MetH uses MeCbl, whereas MetE has no cobamide requirement for its activity, but its expression is regulated by Cbl via a riboswitch (16, 37). Studies have demonstrated that a *metE* deletion mutant of *Mtb* is viable *in vitro* only with CNCbl or L-methionine supplementation (16). This observation led to the interpretation that *Mtb* can transport and convert exogenous CNCbl but cannot synthesize a biologically active Cbl form *de novo* under standard *in vitro* laboratory conditions. In contrast, a *metE*-deficient strain of *Mycobacterium smegmatis* does not require cobamide supplementation owing to its capacity for endogenous cobamide biosynthesis (38). However, simultaneous disruption of *metE* and deletion of *cobK*, a cobamide biosynthesis pathway gene encoding a precorrin-6x reductase, led to cobamide auxotrophy in *M. smegmatis*, which could be resolved through transport and assimilation of exogenous CNCbl (38).

The deletion of *metH* in *Mtb* results in a profound growth defect in media supplemented with CNCbl or cobinamide (39). This phenotype arises because AdoCbl riboswitch-mediated suppression of *metE* expression effectively shuts down all methionine synthase function in a *metH* knockout (16). A similar phenotype is observed in *M. smegmatis*; robust *de novo* cobamide biosynthesis has an equivalent effect by preventing the isolation of a *metH* deletion mutant due to constitutive riboswitch-mediated *metE* suppression (38). This unique phenotype was exploited in a random mutagenesis assay in *Mtb*, which identified Rv1819c as the essential, albeit nonspecific, cobamide transporter, along with other key genes involved in the conversion of CNCbl and cobinamide to the bioactive AdoCbl form (39). These findings underscore the conserved presence of Cbl-sensing riboswitches across mycobacteria, although recent findings have revealed species-specific differences in their sensitivity and ligand specificity (37, 40).

## ROLE OF COBAMIDES IN MYCOBACTERIAL PATHOGENESIS

The role of cobamide in mycobacterial pathogenesis continues to be an open question, partly due to the complex and paradoxical nature of their metabolism in *Mtb*. As noted above, while *Mtb* retains cobamide-requiring enzymes (Table 1) (26, 28), an active transport system (39), and many genes required for cobamide biosynthesis (Fig. 2) (26), the bacilli appear to have lost the capacity for *de novo* synthesis (16–18, 20). This disconnect suggests an evolutionary shift from autonomous cobamide biosynthesis toward dependence on the host.

**Fig 4:**
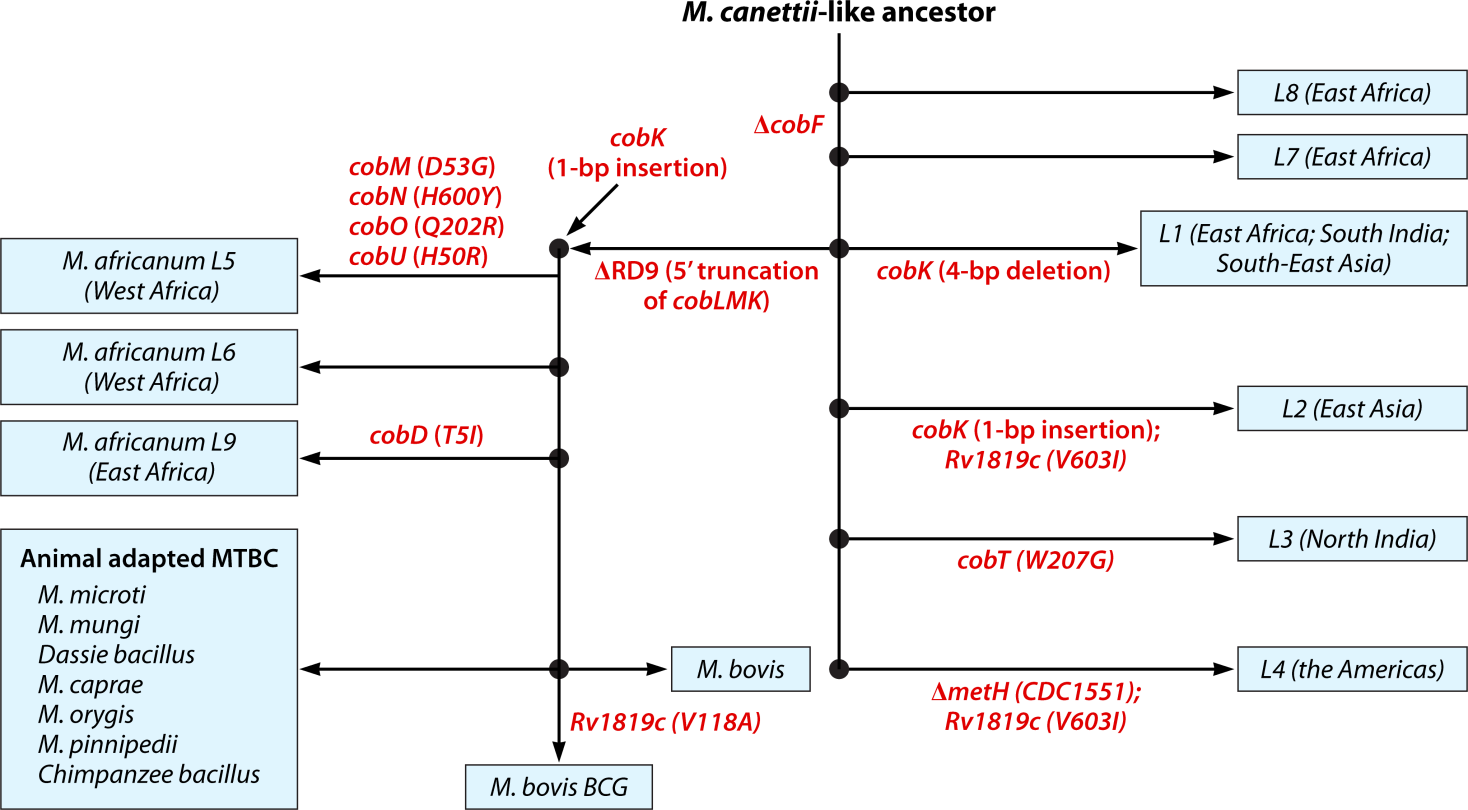
Mutations affecting *de novo* cobamide biosynthesis and metabolism during the evolution of the *M. tuberculosis* complex (MTBC). The figure illustrates phylogenetic relationships within the MTBC and maps of key mutations in cobamide-related genes that occurred during the evolution from a *M. canettii*-like ancestor to the modern human- and animal-adapted lineages. Mutations (indicated by red text) include truncations (RD9; *cobLMK*), deletions (*cobF)*, insertions, and amino acid substitutions in genes involved in cobamide biosynthesis (*cobN, cobO, cobU, cobD, cobB, cobT*), utilisation (*metH*) and processing/uptake (*Rv1314c, Rv1819c*). These lineage-specific disruptions suggest progressive loss or functional divergence of the *de novo* cobamide synthesis pathway, with implications for host adaptation and metabolic remodelling in different geographic contexts. The geographic regions listed in parentheses indicate the areas where the respective MTBC lineages are most distributed. Evolutionary relationships and mutational insights are based on data from 18, 27, 43–46.

Comparative phylogenomics has revealed patterns of conservation, mutation, and loss within the cobamide biosynthetic pathway across mycobacterial species. *Mycobacterium canettii*, the progenitor of the *M. tuberculosis* complex (MTBC), retains a complete and functional cobamide biosynthesis pathway (18, 41). In contrast, all MTBC members, including *Mtb*, show progressive disruptions to this pathway, consistent with reductive evolution and gene loss (Fig. 4) (17, 18). One of the most notable disruptions is the deletion of *cobF*, encoding a precorrin-6a synthase required for cobamide synthesis, in all MTBC lineages except Lineage 8 (L8), where it is retained (Fig. 4) (27). Additional deletions, such as RD9 (region of difference 9), the absence of which is a key distinguishing feature of *Mycobacterium africanum* and the animal-adapted MTBC strains from the other human-adapted lineages, caused a 5′ truncation of *cobL*, which occurs in an operon with *cobM* and *cobK*, potentially disrupting the function of all three genes (Fig. 4) (42). Numerous mutations in at least 10 of 16 core cobamide biosynthesis genes further impair the pathway (Fig. 4) (18, 26). For instance, Minias et al. (26) reported a *cobB* frameshift mutation in ~30% of clinical isolates, along with a G979C mutation in *cobL* and a truncated *metH* allele in CDC1551 among ~1% of the population. Loss-of-function mutations in *nrdZ*, *Rv2067c* (predicted replacement of *cobF*), and *Rv2228c* (predicted α-ribazole phosphatase), as well as a two-base pair deletion upstream of *hemY*, have also been observed. In lineage 5, CobM D53G, CobN H600Y, CobO Q202R, and CobU H50R amino acid substitutions, along with 17 single-nucleotide variants likely to truncate or impair CobN, further illustrate lineage-specific inactivation patterns (Fig. 4) (28).

These genomic alterations support a model of stepwise erosion of the cobamide biosynthetic pathway, shaped by lineage-specific adaptations to a host-dependent lifestyle. This pattern distinguishes modern lineages (L2–L4) from the more ancestral L1, L5, and L6, reflecting relaxed selective pressure for cobamide synthesis, where the cofactor (or its precursors) may be sporadically available from the host.

In contrast, non-tuberculous mycobacteria (NTM) such as *M. smegmatis*, *Mycobacterium marinum*, and *Mycobacterium kansasii* retain intact cobamide biosynthetic pathways and *de novo* synthesis (17, 38, 47, 48). The presence of *cobF* and other biosynthetic genes in NTM, along with their larger genomes and environmental versatility, suggests that cobamide synthesis correlates with metabolic independence and ecological adaptability. The contrast between NTM and MTBC genomes reinforces the view that cobamide loss in MTBC reflects adaptation to an intracellular, host-dependent niche.

A potential codicil is the retention of *cobF* in L8, which suggests that ancestral lineages may have resisted host defenses via partial cobamide synthesis, a capacity lost in modern strains. Whether this represents a retained virulence mechanism or a lost contingency imposed by host pressures remains to be experimentally resolved (18, 26).

**Fig 5:**
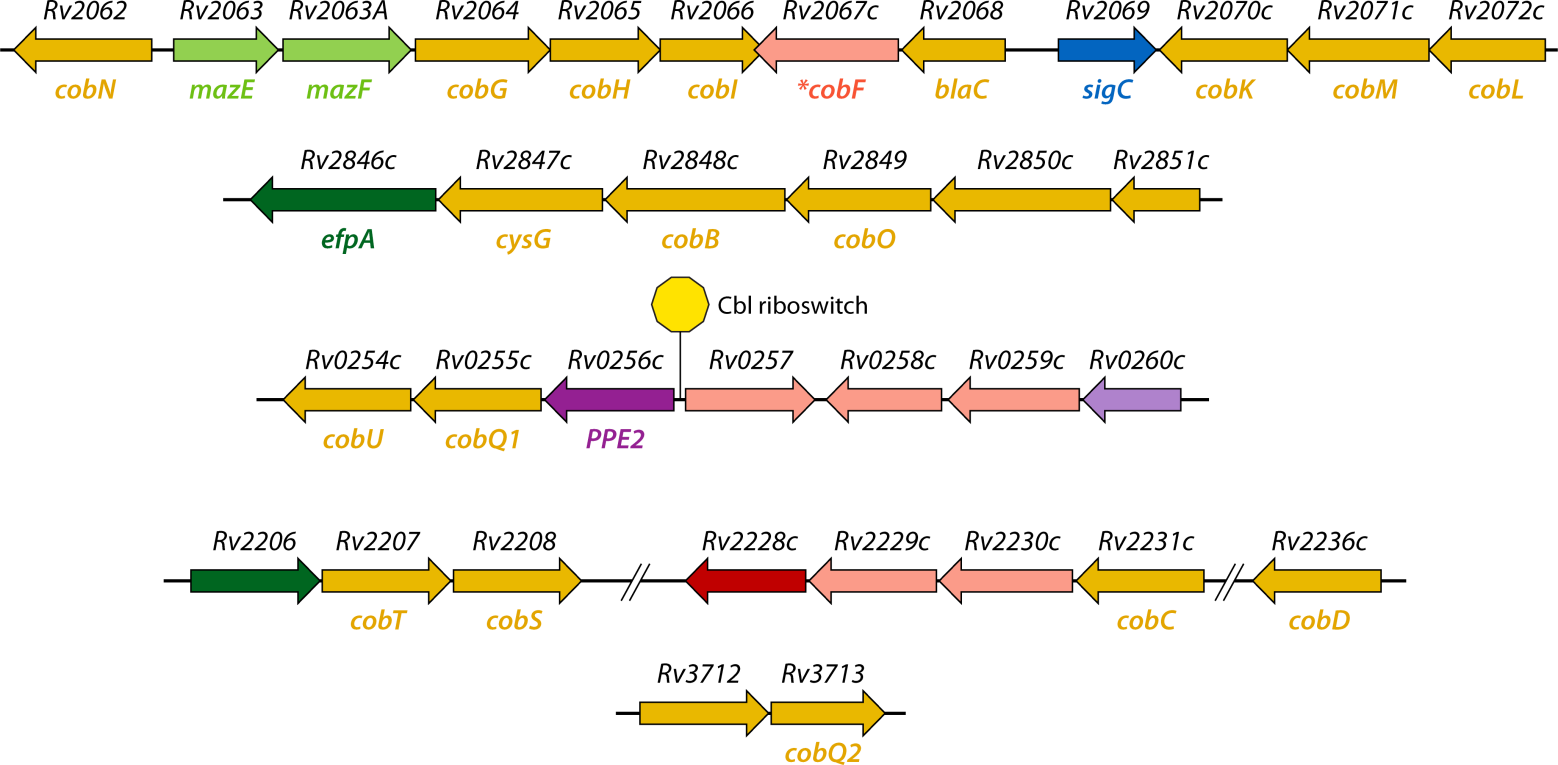
Organization of predicted cobamide biosynthetic genes in *Mtb*. Cobamide-related genes in *Mtb* H37Rv are distributed across five discrete genomic regions. The location of the Cbl riboswitch upstream of *ppe2* is indicated by an orange circle. *Canonical *cobF* is absent in H37Rv but is present in some members of the *Mtb* complex, in smooth tubercle bacilli (41; DOI: 10.1038/ng.2517), and in *M. smegmatis*. Rv2067c has been proposed as a non-orthologous replacement for *cobF* (50).

Despite widespread cobamide biosynthetic disruption in MTBC, experimental studies have explored whether residual synthesis occurs under stress. Minias et al. (17) used enzyme-linked immunosorbent assay (ELISA) based assays to measure intracellular cobamides across multiple growth phases, including logarithmic, stationary, acidified, starved, and hypoxic conditions, and with or without precursor supplementation. In all cases, cobamide levels remained below detection; however, RNA-seq revealed stable transcription of cobamide biosynthetic genes (e.g., *cobA*, *cobG*, and *cobIJ*), suggesting possible post-transcriptional or enzymatic bottlenecks. Similarly, Ignatov et al. (49) observed that, under potassium-deprived, dormancy-inducing conditions, the only group of genes that showed statistically significant upregulation comprised cobamide-synthesizing enzymes, including the cobalamin biosynthetic operon *cobJHKLM* (Fig. 5); again, however, these authors found no direct evidence of cobamide production. Together, these results reinforce the idea that the pathway is transcriptionally responsive but functionally incomplete.

Despite the apparent loss of cobamide synthesis, cobamide-related genes remain under strong purifying selection (26). In their analysis of nearly 4,000 clinical genomes, Minias et al. (26) found that genes involved in cobamide transport and utilization had dN/dS ratios well below 1. This suggests tightly preserved functional roles, potentially related to uptake, salvage, or fine-tuning cobamide utilization when host-derived cobamides or precursors are intermittently available.

Several studies suggest that *Mtb* may access and utilize host-derived cobamides. Although 5%–30% of measurable dietary Cbl may consist of microbiologically active but incomplete precursors (51, 52), intrinsic factor-mediated absorption is highly selective for intact Cbl, and there is no evidence that analog forms are absorbed through this pathway. Non-absorbed dietary Cbl reaches the large intestine, where microbial communities may remodel it into variant forms; however, these analogs are not known to be absorbed into systemic circulation. Reports of Cbl analogs detected in human plasma (53–55) and rabbit models (56, 57) are difficult to interpret, as most were identified through binding assays and may represent modified or degraded forms of Cbl rather than structurally complete or biologically active cobamides. Importantly, these analogs are unlikely to bind transcobalamin and may instead associate with haptocorrin, limiting their bioavailability. Furthermore, there is currently no direct evidence that such analogs are absorbed from the large intestine or circulate in a form usable by *Mtb*. Therefore, the significance of these analogs in the context of human infection, and whether *Mtb* can salvage and remodel them into functional forms, remains to be experimentally demonstrated. Cbl remains the predominant and functionally relevant cobamide in mammalian systems, and *Mtb* likely acquires and utilizes this form during infection.

Campos-Pardos et al. (18) showed that *Mtb* exhibits reduced fitness in Cbl-deficient hosts, whereas *M. canettii* does not. The reliance of *Mtb* on the MeCbl-requiring MetH during infection further highlights the functional dependency on host-derived Cbl. Thus, despite the reported inability of *Mtb* to synthesize cobamides, these cofactors appear to remain central to its pathogenesis.

Altogether, genomic, experimental, and evolutionary evidence supports a coherent model in which cobamides remain essential to *Mtb* through a shift from biosynthesis to salvaging. The retention and purifying selection of cobamide-related genes and their active transcription under stress conditions suggest that these genetic elements have been repurposed for the regulated uptake and metabolic integration of host-derived cobamides. This metabolic transition parallels broader genome streamlining that transformed *Mtb* from an environmentally flexible ancestor like *M. canettii* into a specialized human pathogen. Notably, reduced virulence under cobamide-limited *in vivo* conditions highlights cobamide acquisition as a determinant of pathogenic fitness.

## HOST IMMUNE PRESSURE AS AN EVOLUTIONARY FORCE

Comparative genomic and functional studies have shown that host immune pressures have profoundly shaped the evolutionary trajectory of *Mtb*, producing variants better equipped to endure nutrient restriction, oxidative stress, and antimicrobial defenses encountered within host macrophages (58, 59). In response, *Mtb* has developed strategies to persist intracellularly by adapting its metabolism to host-imposed stresses. These metabolic adaptations likely reflect evolutionary responses to immune-mediated selective pressures, including a shift toward dependence on host-derived cobamides (18). Specific lineage-level adaptations, such as mutations in secreted effectors that alter macrophage behavior, deletions that enhance hypoxia tolerance, and differential sensitivity to metabolic stressors, further illustrate how *Mtb* has diversified under immune-driven selection (58, 59).

Thus, *Mtb* shows significant metabolic flexibility, enabling it to thrive within diverse and often hostile host environments. During infection, it preferentially catabolizes host-derived fatty acids and cholesterol, relying on the glyoxylate shunt, methylcitrate cycle, and methylmalonyl pathway to generate energy and detoxify propionyl-CoA (23, 60). These pathways converge on key enzymes whose activity is tightly linked to central carbon metabolism, including AdoCbl-dependent MCM (Fig. 3).

Among the host-imposed metabolic constraints that challenge this flexibility is itaconate, an antimicrobial and immunomodulatory metabolite produced by activated macrophages via cis-aconitate decarboxylase, encoded by immune-responsive gene 1 (*Irg1*) (32). *Irg1* is upregulated during *Mtb* infection, and *Irg1*-deficient mice exhibit increased susceptibility and greater lung pathology, underscoring the role of itaconate in host antimycobacterial defenses (31, 61). Amplifying this response is the aryl hydrocarbon receptor (AhR), a transcriptional regulator activated during infection and inflammation, enhancing *Irg1* expression and boosting itaconate production (62). The accumulation of itaconate and its derivative, itaconyl-CoA, inactivates the AdoCbl-dependent MCM by forming a stable biradical with AdoCbl (29). This mechanism of inhibition presumably induces localized AdoCbl depletion, though whether this occurs within the host macrophage, in the intracellular *Mtb* bacillus, or both, remains to be determined. Nonetheless, the inhibition of *Mtb* MCM may reduce the pool of functional Cbl available for bacilli, potentially restricting growth within macrophages and illustrating how host cells might leverage metabolic disruption to restrict nutrient access to intracellular pathogens. Interestingly, CNCbl has been identified as a natural AhR antagonist (63), suggesting a feedback mechanism whereby host Cbl levels could modulate AhR activity and, consequently, itaconate production.

Itaconate exerts its antimicrobial activity by covalently modifying and inhibiting multiple bacterial enzymes involved in central metabolism (Fig. 3). In *Mtb*, this includes isocitrate lyase, a bifunctional enzyme critical for both the glyoxylate shunt and the methylcitrate cycle, which are essential for fatty acid utilization and propionyl-CoA detoxification during intracellular growth (20, 31, 64–67). Itaconyl-CoA-mediated inhibition of MCM blocks essential lipid-processing and propionate detoxification pathways, creating a significant metabolic bottleneck. Recent studies have identified additional itaconate-sensitive targets such as aldolase and inosine monophosphate dehydrogenase (GuaB2) (30), indicating that itaconate disrupts multiple layers of *Mtb* metabolism beyond lipid catabolism. These findings highlight the importance of cobamide-requiring enzymes for *in vivo* survival of *Mtb* and reinforce why selective pressures could favor the retention and possible repurposing of cobamide-related genes involved in cofactor salvage, transport, or regulatory roles, despite the apparent loss of *de novo* synthesis capacity.

Given these profound metabolic challenges, it is not surprising that *Mtb* has evolved targeted countermeasures to detoxify itaconate and repurpose it as a nutrient source. Detoxification begins with itaconate:succinyl-CoA transferase, which converts itaconate into itaconyl-CoA, followed by itaconyl-CoA hydratase and citramalyl-CoA lyase (Rv2498c), which degrade citramalate into acetyl-CoA and pyruvate, which feed directly into the tricarboxylic acid (TCA) cycle (30, 68). Thus, *Mtb* converts a host-derived antimicrobial metabolite into a metabolic substrate, demonstrating extraordinary flexibility. The pathway’s importance is evident from the impaired growth of mutants lacking these enzymes in macrophages and *in vivo*, underscoring a critical role in detoxification and metabolic adaptation (68). This dual nature of itaconate, as both antimycobacterial effector and metabolic substrate, has likely shaped the adaptive evolution of *Mtb*, allowing the bacterium to circumvent immune restriction while salvaging carbon and preserving cobamide-dependent activities.

In addition to metabolite-driven antimicrobial strategies such as itaconate, host genetic variability further shapes the metabolic landscape encountered by *Mtb*. One example is the mitochondrial enzyme, CLYBL (Citrate Lyase Beta Like), involved in Cbl metabolism and itaconate detoxification in the host (33). CLYBL catalyzes the conversion of (*S*)-citramalyl-CoA, a downstream product of itaconate metabolism, into acetyl-CoA and pyruvate (33), preventing the accumulation of toxic intermediates like itaconyl-CoA that can inhibit the host’s own MCM.

Thus, CLYBL acts as a metabolic safeguard, protecting host mitochondrial function and preserving the activity of the AdoCbl-dependent enzyme. A common loss-of-function polymorphism in the *CLYBL* gene (*rs41281112*), present in ~5% of the population, introduces a premature stop codon that reduces enzyme function and is associated with lower systemic Cbl levels (69–71).

This deficiency is thought to arise from the accumulation of itaconyl-CoA, which irreversibly inactivates AdoCbl and disrupts mitochondrial Cbl metabolism (29, 33). CLYBL polymorphisms may impose an additional layer of metabolic constraints on intracellular pathogens by limiting Cbl availability within macrophages. Since *Mtb* likely relies on host-derived cobamide for essential metabolic functions like propionate detoxification via MCM, reduced intracellular Cbl pools could restrict bacterial survival.

From the host perspective, this might enhance immune-mediated nutrient restriction. However, excessive Cbl depletion could impair host cell metabolism or exacerbate inflammation. Despite this mechanistic plausibility, there is presently no direct genetic evidence connecting CLYBL polymorphisms with human susceptibility toward *Mtb* infection or TB progression. Clarifying this relationship will require focused genetic and functional analyses.

Such host-driven genetic adaptations likely exert variable pressures on bacterial cobamide acquisition strategies, potentially selecting for more efficient salvaging mechanisms in populations with limited cobamide availability. Host genetic variability and, in turn, Cbl status add another layer of complexity to the nutritional immunity landscape, further shaping the evolutionary trajectory of *Mtb*.

## TRANSPORT AND ASSIMILATION OF COBAMIDES AND COBAMIDE PRECURSORS

### Transport

Pathogenic mycobacteria universally lack the two major cobamide transport systems found in gram-negative bacteria: the canonical import system, consisting of the BtuB outer membrane receptor energized by TonB-ExbBD, together with the BtuFCD inner membrane ABC transporter, and the energy coupling factor (ECF) transporters, which belong to a distinct class of inner membrane uptake systems (72–78). Some NTMs like *Mycobacterium abscessus* and *M. smegmatis* encode predicted *btuFCD* analogs (79), which show strong topological similarity (>60%) to the canonical BtuFCD proteins despite their low sequence identity (~20%). While this indicates a conserved fold, its functional significance for understanding cobamide transport in other mycobacteria including *Mtb* remains uncertain. To date, no mycobacterium is known to encode an outer membrane transporter for cobamide import, nor any constituent of the ECF-type transporters.

The ATP-dependent ABC transporter Rv1819c remains the sole assigned importer for CNCbl, AdoCbl, and cobinamide (39, 80). However, since Rv1819c displays substrate promiscuity and the potential for export (81), there are unresolved mechanisms controlling substrate recognition and transport directionality, as well as likely cooperation of Rv1819c with other unidentified protein partners. Moreover, the hydrophobicity of the mycobacterial cell likely precludes passive cobamide diffusion, suggesting active import. This is further supported by the essentiality of Rv1819c ATP hydrolysis activity, demonstrated by an inactivating Walker B mutant E576G (80).

The role of Rv1819c in *Mtb* pathogenicity appears to be stage specific in the TB disease spectrum. While Rv1819c is dispensable for acute infection in mice, it is critical for chronic persistence (82). This may indicate that *Mtb* accesses host-derived cobamides differently across infection phases, with Rv1819c acting as a low-efficiency route for salvaged cobamides in phagolysosomes during early infection (83–85). In latency, *Mtb* might access host Cbl that has been released from transcobalamin following lysosomal degradation in nutrient-poor monocytes (86), via uncharacterized mechanisms (84, 87). By analogy, *Mtb* could employ mechanisms akin to Bacteroides BtuG-mediated stripping of intrinsic factor (88). However, these mechanisms remain speculative, and high-resolution tools, such as single-molecule tracking or live-cell imaging, are needed to uncover the spatial and temporal details of uptake dynamics.

### Adenosylation

Following uptake, CNCbl or cobinamide must be adenosylated to form AdoCbl, the active cofactor (Fig. 3). *Mtb* encodes two putative adenosyltransferases for this function, Rv1314c (PduO-type adenosyltransferase), which genetic evidence suggests is the dominant adenosyltransferase (39), and CobO, which has an unresolved function. Therefore, Rv1314c is probably the primary enzyme for generating AdoCbl. Structural studies have shown that Rv1314c not only synthesizes AdoCbl but also chaperones it to MCM, with its DMB tail orchestrating cofactor transfer (89, 90). Remarkably, Rv1314c can also catalyze sacrificial Co-C bond homolysis, which is a reversal of its synthetic role.

Under laboratory conditions, growth in CNCbl- or dicyanocobinamide-supplemented media may necessitate decyanation within the bacterial cells as a pre-processing step (91, 92). After decyanation, the resulting species are converted into the base-off form (e.g., cob[II]alamin), which can be adenosylated to AdoCbl or methylated to MeCbl, the final active forms required for metabolism (92). In some bacteria, BtuM functions as both a transporter and a decyanating enzyme (91, 93–95). CNCbl binding to BtuM induces the base-off state, crucial for subsequent decyanation and transport (93). *Mtb* lacks a BtuM homolog, and the presence of a decyanation step in this organism remains unconfirmed (Fig. 3).

### Lower ligand assembly and cobamide diversity

The identity and assembly of the lower base are critical to cobamide diversity. In *Mtb*, the lower base is probably DMB, assembled during the late stages of the Cbl biosynthesis pathway. While *Mtb* encodes *Rv0306*, annotated as a BluB homolog based on sequence similarity to known DMB synthases from other bacteria (96–98), its enzymatic activity has not been empirically confirmed. Moreover, a dedicated flavin mononucleotide (FMN) reductase to supply reduced flavin (FMNH₂) as the substrate for BluB activity has not been definitively characterized in *Mtb*. The CobT phosphoribosyltransferase catalyzes the condensation of DMB with nicotinate mononucleotide (NaMN) to form α-ribazole-5’-phosphate (α-RP; Fig. 2) (13). Rv0306 has a predicted functional association with CobT in the STRING database (99), further supporting CobT involvement in lower ligand assembly.

Interestingly, our unpublished data suggest that *Mtb* CobT may be partially dispensable for lower ligand synthesis and cobinamide salvage, raising the possibility of bypass via CobB. Studies in *Salmonella typhimurium* have shown that CobB, a sirtuin deacetylase, can exhibit CobT-like activity (13, 100, 101). Additionally, CobT may use NAD^+^ instead of NaMN to generate α−5,6-DMB adenine dinucleotide, which could be cleaved to form α-RP (102). Whether *Mtb* employs similar bypass mechanisms is unknown, and the full extent of cobamide diversity in this organism has yet to be elucidated. This highlights the need to better understand how *Mtb* assembles and remodels cobamides, particularly given the potential regulatory and functional impact of lower ligand variation.

## REGULATION BY COBALAMIN

### Systemic metabolic rewiring under cobalamin control

RNA-seq analysis has revealed that Cbl exerts broad regulatory effects in *Mtb*, supporting its varied roles in *Mtb* physiology and pathogenicity (103). These Cbl-mediated transcriptional shifts include repression of the PrpR operon (methyl citrate cycle) and activation of virulence lipid synthesis (*aprA*) and ESX-1-associated genes (EspR regulon), spanning metabolic networks linked to stress responses, cell wall integrity, antibiotic resistance, and latency (103). These Cbl-responsive regulons suggest novel regulatory nodes for future investigation.

### Cobalamin-sensing via riboswitches

In *Mtb*, as in most bacteria, Cbl has been shown to post-transcriptionally regulate expression of genes involved in Cbl metabolism via Cbl-sensing riboswitches. Cbl riboswitches are widespread in prokaryotes and share conserved features, such as a ligand-binding core, a four-way junction, a consensus “B_12_ box,” and a “kissing loop” (KL) tertiary element (104–107). Interestingly, they exhibit distinct selectivities toward different Cbl isoforms, leading to their classification into class I, IIa, or IIb (107). Class I and IIb riboswitches preferentially bind AdoCbl, whereas class IIa riboswitches favor MeCbl and hydroxocobalamin (OHCbl). Some riboswitches, however, bind a broader range of corrinoids (107).

**Fig 6:**
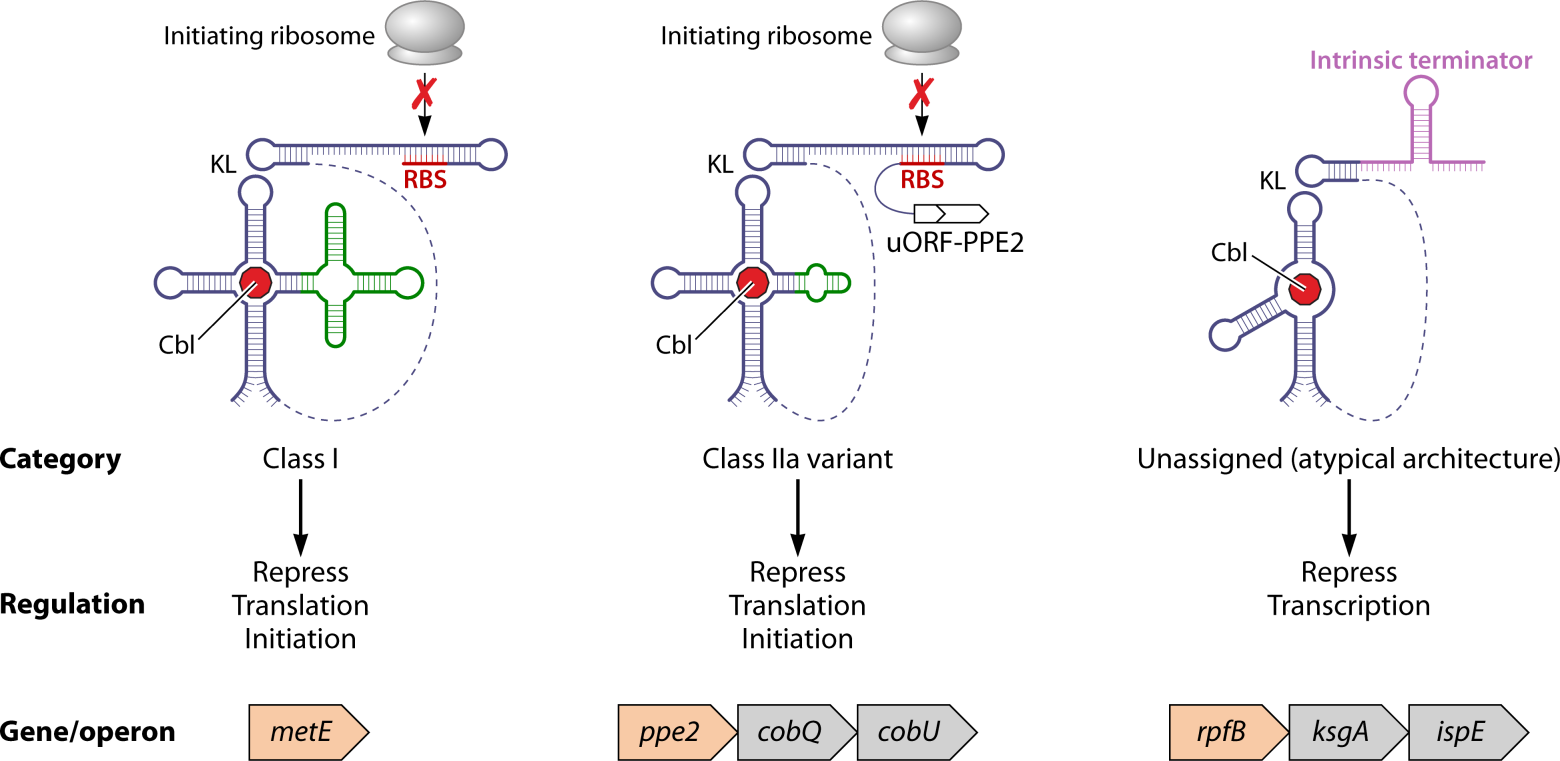
A schematic illustrating the structural and regulatory diversity of three distinct Cbl-sensing riboswitches and their mechanisms of gene regulation in *Mtb*. The *metE* riboswitch (class I) represses translation initiation via AdoCbl-dependent occlusion of the ribosome binding site (RBS) and features a canonical four-way junction with a conserved KL pseudoknot. The *ppe2* riboswitch (class IIa variant) also contains a four-way junction and KL motif but regulates gene expression through a uORF-dependent translational relay. The atypical *rpfB* riboswitch lacks both the four-way junction and canonical “B_12_ box” and is proposed to mediate low-affinity AdoCbl binding via a three-way junction, triggering transcriptional termination through an intrinsic terminator.

Recent studies have provided insight into how structural and mechanistic diversity of Cbl riboswitches in *Mtb* (Fig. 6) enables pathogen adaptation to host metabolic constraints (37, 85). Unlike most bacteria, in which Cbl riboswitches primarily regulate genes with obvious involvement in cobamide biosynthesis, utilization, or transport, *Mtb* encodes three distinct switches (Fig. 6) and appears to have co-opted at least one to control virulence and persistence, reflecting a specialized evolutionary trajectory (85).

Two of the *Mtb* Cbl riboswitches were discovered through comparative genomics. The first to be investigated experimentally controls *metE*, encoding the Cbl-independent methionine synthase (16). The second controls a tricistronic operon containing PPE2, an immunomodulatory protein initially hypothesized to function as a cobalt transporter owing to predicted transmembrane domains, and CobQ and CobU, remnants of the cobamide biosynthetic pathway (Fig. 5) (50). Emerging evidence now points to other roles for PPE2 in host modulation, for example, suppression of nitric oxide production (108, 109), inhibition of reactive oxygen species generation (110), and altering myeloid cell differentiation (111). The operonic association of *ppe2* with *cobQ-cobU* and the presence of a Cbl riboswitch at this genomic locus are not strongly conserved across mycobacteria. For example, *cobQ*/*cobU* are pseudogenes in *Mycobacterium leprae*, while in *M. marinum* and *Mycobacterium avium*, *ppe2* is disassociated from the *cobQ-cobU* operon.

The *metE* switch was initially thought to control gene expression at the transcriptional level (16). Later work identified it as a probable class I switch employing dual mechanisms: AdoCbl-dependent translational repression of the *metE* translation initiation region coupled with Rho-dependent termination (37). Its modest affinity (Kd ~20 µM) and AdoCbl specificity imply optimization for sensing host-derived adenosylated Cbl. Additionally, a translatable uORF embedded in the *metE* leader sequence further influences expression, although the precise nature of uORF-mediated regulation remains unclear.

The *ppe2* switch is distinguished by its compact architecture and its regulation of PPE2 expression via a translational relay through an uORF (uPPE2), which is essential for initiating translation of the downstream PPE2 gene (37). Although the two reading frames are distinct, *uPPE2* and *PPE2* share a 4-nucleotide overlap, where the stop codon of *uPPE2* (UGA) and the start codon of *PPE2* (AUG) form the overlapping tetranucleotide *AUGA* (37). This arrangement enables a translation termination–reinitiation mechanism, occasionally accompanied by stop codon readthrough, resulting in *uPPE2–PPE2* fusion proteins (37). In contrast to the *metE* switch, the *ppe2* riboswitch displays hallmarks of class IIa riboswitches, particularly its promiscuous binding to a range of Cbl isoforms (37).

### Cobalamin-sensing link to resuscitation from dormancy

A third, functionally uncharacterized Cbl riboswitch was recently identified upstream of the *rpfB* operon (*rpfB-ksgA-ispE*), involved in bacillary resuscitation (*rpfB*), ribosomal maturation (*ksgA*), and peptidoglycan synthesis (*ispE*) (85, 112). These processes are not directly tied to canonical cobamide metabolism, making the regulatory placement of this riboswitch particularly intriguing and suggesting a potentially unconventional role for Cbl in non-replicating persistence. Unlike the other two *Mtb* riboswitches, the *rpfB* switch controls gene expression at the level of transcription via an intrinsic terminator, a mechanism that is exceptionally rare among mycobacteria and has only been identified within the MTBC strains (112). While it remains unconfirmed, this riboswitch challenges established assumptions about Cbl riboswitch architecture and function, as it apparently lacks both the conserved “B_12_ box” and four-way junction typical of canonical Cbl switches (85). Preliminary data shows that the *rpfB* switch binds AdoCbl with only low affinity (millimolar range), through a binding pocket possibly situated in a three-way junction (Fig. 6) (85).

Together, the three distinct Cbl riboswitches illustrate how *Mtb* might have adapted a conserved regulatory scaffold to support its pathogenic lifestyle. Their structural and mechanistic divergence, particularly the use of a minimal architecture of the *rpfB* element, raises compelling questions about the evolutionary pressures that shape riboswitch diversity in *Mtb*. One emerging hypothesis is that the *metE* switch reflects metabolic thrift, the *ppe2* switch integrates virulence and residual cofactor metabolism, and the *rpfB* switch links host environmental sensing to non-replicating persistence. It remains unclear, however, whether riboswitch activity is modulated by distinct regulatory demands across infection stages, or how these elements might be harnessed for therapeutic purposes, such as targeted gene control or metabolic disruption.

## TARGETING B_12_ METABOLISM FOR TB THERAPY

Although cobamide-requiring enzymes are non-essential for *Mtb* growth under standard laboratory conditions, their retention suggests that they may confer adaptive advantages in host environments, where purifying selection likely maintains their function (26, 28, 41). This apparent reliance highlights cobamide metabolism as a potential therapeutic target, although optimism is tempered by several fundamental challenges. Despite the vulnerability implied by genomic erosion of cobamide biosynthesis pathways, no enzymes in this pathway have been successfully inhibited to date. Further complicating this, essential genes such as *hemD/cysG*, *cobC*, and *cobQ2* (98–101) participate in multiple cellular processes beyond cobamide metabolism, so their inhibition could disrupt both cobamide-dependent and unrelated metabolism (14). Technical barriers also limit therapeutic development, particularly the absence of a dedicated outer membrane transport system analogous to the BtuB-BtuFCD machinery exploited for the delivery of cobamide-drug conjugates in other bacteria (29, 113–118). Moreover, even if Rv1819c was the sole cobamide transporter, its broad substrate specificity raises concerns about potential off-target effects.

While direct targeting of cobamide pathways remains challenging, emerging strategies suggest that indirect disruption of cobamide metabolism may offer therapeutic advantages. One such approach involves the “methylfolate trap,” where Cbl deficiency induces a metabolic blockade in folate cycling, sensitizing *Mtb* to sulfonamides (119). Thus, dual inhibition of the folate pathway and Cbl availability could be synthetically lethal and might circumvent conventional resistance, though this strategy still requires *in vivo* validation. Additionally, Cbl has been shown to upregulate the *iniBAC* operon, which modifies cell wall structure in response to drug pressure (120). Limiting Cbl availability could therefore potentiate existing antibiotics by preventing adaptive resistance. However, it remains unclear whether host-derived Cbl levels are sufficient to sustain this regulatory effect.

## EXTRACTION, DETECTION, AND ANALYSIS OF COBAMIDES IN *MTB*

The extraction of cobamides from mycobacteria requires careful consideration owing to the unique properties of their cell walls. Standard protocols involve mechanical cell disruption (e.g., bead beating or sonication), resuspension in acetate buffer (pH 4.5), boiling with potassium cyanide, cooling, and centrifugation (98). Cyanide treatment stabilizes intracellular cobamide by replacing the upper ligand with a cyano group, thereby converting oxygen-sensitive forms into their corresponding cyano forms, such as CNCbl (38, 121). This circumvents the need for anaerobic conditions otherwise required to preserve redox-sensitive species such as cob(II)alamin (121). Subsequent purification via solid-phase extraction (C18 columns) effectively removes abundant lipids from mycobacterial lysates prior to downstream analysis (98).

Liquid chromatography-tandem mass spectrometry with multiple reaction monitoring remains the gold standard for cobamide detection due to its unparalleled specificity in distinguishing physiologically relevant isoforms (38, 98, 122). This is particularly important for differentiating “true” Cbl from pseudo-Cbl in clinical isolates. For high-throughput quantification, adapted immunoassays, including ELISA (17) and clinical-grade electrochemiluminescence platforms (18), are widely used in clinical practice but have recognized limitations in sensitivity and specificity for diagnosing deficiency (123).

Microbiological assays exploiting cobamide-dependent growth have provided foundational insights into the utilization of the cofactor by *Mtb* (16, 38, 39, 48, 120, 122). Fluorescent reporters and riboswitch-based biosensors have been adapted to study cobamide-related metabolism in *Mtb* and *M. marinum* (48, 120, 124). Whole-cell Cbl biosensors in *E. coli*, including one in which AdoCbl binding to a surface-displayed sensor protein, CarH, promotes tetramerization and induces bacterial agglutination (125), could inspire analogous *Mtb* tools to track Cbl competition in host niches. As these methodologies advance, they promise new insights into how *Mtb* exploits available host-derived cobamides and precursors, revealing its metabolic adaptability.

## CONCLUSION

Recent advances, spanning genomics, structural biology, host-pathogen interactions, and regulatory studies, have illuminated how cobamides shape *Mtb* physiology. Evidence indicates that host-derived metabolites and immune pressures intersect with cobamide-dependent pathways to constrain or rewire *Mtb* metabolism during infection. The retention of cobamide-related genes under purifying selection, despite biosynthetic erosion, underscores their repurposing for regulated uptake, enzymatic function, and adaptive gene control (26). Mechanistically, *Mtb* salvages host-derived corrinoids via a non-canonical ABC transporter and a PduO-type adenosyltransferase (14, 39, 89), while riboswitches with distinct ligand specificities modulate methionine synthesis, cobalt transport, and resuscitation from dormancy (16, 37, 85). These elements form a finely tuned system responsive to intracellular cues and host constraints.

Although no experimental evidence supports *de novo* cobamide synthesis in *Mtb* under standard conditions, active transcription of biosynthetic genes under stress conditions such as hypoxia, nutrient limitation, or dormancy has been observed (26, 49). These findings suggest retained regulatory responsiveness, not functional synthesis. However, we cannot rule out that these responses reflect vestigial regulation or a latent capacity for synthesis under untested conditions.

Several key questions persist: How does *Mtb* access host cobamide pools? Can it exploit high-affinity transport systems? What roles do orphan genes like *cobO* play? How do riboswitches discriminate among corrinoid variants?

In conclusion, the paradoxical role of cobamides and their structural diversity in mycobacteria remains an area of open investigation. It is unclear whether *Mtb* can utilize non-canonical cobamides, and if alternative cobamide species might support survival in nutrient-limited or host-associated environments. Understanding how *Mtb* accesses, senses, and integrates cobamides into its physiology, and clarifying the structural and functional diversity of cobamides it can access, could reveal niche-specific adaptations and inform therapeutic strategies targeting cofactor acquisition and remodeling. In this light, cobamide metabolism is not merely a metabolic curiosity but a promising frontier for therapeutic innovation.

## References

[1] Escalante-Semerena JC, Warren MJ. 2008. Biosynthesis and use of cobalamin (B12). EcoSal Plus 3:3. doi:10.1128/ecosalplus.3.6.3.8 26443728

[2] Martens JH, Barg H, Warren MJ, Jahn D. 2002. Microbial production of vitamin B12. Appl Microbiol Biotechnol 58:275–285. doi:10.1007/s00253-001-0902-7 11935176

[3] Warren MJ, Raux E, Schubert HL, Escalante-Semerena JC. 2002. The biosynthesis of adenosylcobalamin (vitamin B12). Nat Prod Rep 19:390–412. doi:10.1039/b108967f 12195810

[4] Raux E, Schubert HL, Roper JM, Wilson KS, Warren MJ. 1999. Vitamin B12: insights into biosynthesis’s mount improbable. Bioorg Chem 27:100–118. doi:10.1006/bioo.1998.1125

[5] Kräutler B. 2005. Vitamin B12: chemistry and biochemistry. Biochem Soc Trans 33:806–810. doi:10.1042/BST0330806 16042603

[6] Matthews RG, Koutmos M, Datta S. 2008. Cobalamin-dependent and cobamide-dependent methyltransferases. Curr Opin Struct Biol 18:658–666. doi:10.1016/j.sbi.2008.11.005 19059104 PMC2639622

[7] Fang H, Kang J, Zhang D. 2017. Microbial production of vitamin B12: a review and future perspectives. Microb Cell Fact 16. doi:10.1186/s12934-017-0631-y PMC5282855 28137297

[8] Shelton AN, Seth EC, Mok KC, Han AW, Jackson SN, Haft DR, Taga ME. 2019. Uneven distribution of cobamide biosynthesis and dependence in bacteria predicted by comparative genomics. ISME J 13:789–804. doi:10.1038/s41396-018-0304-9 30429574 PMC6461909

[9] Zhang Y, Rodionov DA, Gelfand MS, Gladyshev VN. 2009. Comparative genomic analyses of nickel, cobalt and vitamin B12 utilization. BMC Genomics 10. doi:10.1186/1471-2164-10-78 PMC2667541 19208259

[10] Sobczyńska-Malefora A, Smith AD. 2022. Vitamin B-12. Adv Nutr 13:2061–2063. doi:10.1093/advances/nmac030 35348594 PMC9526831

[11] Mascarenhas R, Gouda H, Ruetz M, Banerjee R. 2022. Human B12-dependent enzymes: methionine synthase and methylmalonyl-CoA mutase, p 309–326. In In Methods in Enzymology. Academic Press Inc. 10.1016/bs.mie.2021.12.012 PMC9420401 35589199

[12] Ankar A, Kumar A. 2024. Vitamin B12 Deficiency. StatPearls Publishing, StatPearls [Internet]. Treasure Island (FL). 28722952

[13] Villa EA, Escalante-Semerena JC. 2024. Corrinoid salvaging and cobamide remodeling in bacteria and archaea. J Bacteriol 206:e0028624. doi:10.1128/jb.00286-24 39404452 PMC11580458

[14] Gopinath K, Moosa A, Mizrahi V, Warner DF. 2013. Vitamin B_12_ metabolism in Mycobacterium tuberculosis. Future Microbiol 8:1405–1418. doi:10.2217/fmb.13.113 24199800

[15] Cole ST, Brosch R, Parkhill J, Garnier T, Churcher C, Harris D, Gordon SV, Eiglmeier K, Gas S, Barry CE III, et al.. 1998. Deciphering the biology of Mycobacterium tuberculosis from the complete genome sequence. Nature 393:537–544. doi:10.1038/31159 9634230

[16] Warner DF, Savvi S, Mizrahi V, Dawes SS. 2007. A riboswitch regulates expression of the coenzyme B12-independent methionine synthase in Mycobacterium tuberculosis: implications for differential methionine synthase function in strains H37Rv and CDC1551. J Bacteriol 189:3655–3659. doi:10.1128/JB.00040-07 17307844 PMC1855906

[17] Minias A, Gąsior F, Brzostek A, Jagielski T, Dziadek J. 2021. Cobalamin is present in cells of non-tuberculous mycobacteria, but not in Mycobacterium tuberculosis. Sci Rep 11:12267. doi:10.1038/s41598-021-91430-w 34112827 PMC8192938

[18] Campos-Pardos E, Uranga S, Picó A, Gómez AB, Gonzalo-Asensio J. 2024. Dependency on host vitamin B12 has shaped Mycobacterium tuberculosis complex evolution. Nat Commun 15:2161. doi:10.1038/s41467-024-46449-8 38461302 PMC10924821

[19] Kapopoulou A, Lew JM, Cole ST. 2011. The MycoBrowser portal: a comprehensive and manually annotated resource for mycobacterial genomes. Tuberculosis (Edinb) 91:8–13. doi:10.1016/j.tube.2010.09.006 20980200

[20] Savvi S, Warner DF, Kana BD, McKinney JD, Mizrahi V, Dawes SS. 2008. Functional characterization of a vitamin B12-dependent methylmalonyl pathway in Mycobacterium tuberculosis: implications for propionate metabolism during growth on fatty acids. J Bacteriol 190:3886–3895. doi:10.1128/JB.01767-07 18375549 PMC2395058

[21] Lee W, VanderVen BC, Fahey RJ, Russell DG. 2013. Intracellular Mycobacterium tuberculosis exploits host-derived fatty acids to limit metabolic stress. Journal of Biological Chemistry 288:6788–6800. doi:10.1074/jbc.M112.445056 23306194 PMC3591590

[22] Muñoz‐Elías EJ, Upton AM, Cherian J, McKinney JD. 2006. Role of the methylcitrate cycle in Mycobacterium tuberculosis metabolism, intracellular growth, and virulence . Mol Microbiol 60:1109–1122. doi:10.1111/j.1365-2958.2006.05155.x 16689789

[23] Griffin JE, Pandey AK, Gilmore SA, Mizrahi V, Mckinney JD, Bertozzi CR, Sassetti CM. 2012. Cholesterol catabolism by Mycobacterium tuberculosis requires transcriptional and metabolic adaptations. Chemistry & Biology 19:218–227. doi:10.1016/j.chembiol.2011.12.016 22365605 PMC3292763

[24] Dawes SS, Warner DF, Tsenova L, Timm J, McKinney JD, Kaplan G, Rubin H, Mizrahi V. 2003. Ribonucleotide reduction in Mycobacterium tuberculosis: function and expression of genes encoding class Ib and class II ribonucleotide reductases. Infect Immun 71:6124–6131. doi:10.1128/IAI.71.11.6124-6131.2003 14573627 PMC219568

[25] Mowa MB, Warner DF, Kaplan G, Kana BD, Mizrahi V. 2009. Function and regulation of class I ribonucleotide reductase-encoding genes in mycobacteria. J Bacteriol 191:985–995. doi:10.1128/JB.01409-08 19028890 PMC2632068

[26] Minias A, Minias P, Czubat B, Dziadek J. 2018. Purifying selective pressure suggests the functionality of a vitamin B12 biosynthesis pathway in a global population of Mycobacterium tuberculosis. Genome Biol Evol 10:2326–2337. doi:10.1093/gbe/evy153 30060031 PMC6363050

[27] Ngabonziza JCS, Loiseau C, Marceau M, Jouet A, Menardo F, Tzfadia O, Antoine R, Niyigena EB, Mulders W, Fissette K, Diels M, Gaudin C. 2020. A sister lineage of the Mycobacterium tuberculosis complex discovered in the African great lakes region. Nat Commun 11:2917. doi:10.1038/s41467-020-16626-6 32518235 PMC7283319

[28] Young DB, Comas I, de Carvalho LPS. 2015. Phylogenetic analysis of vitamin B12-related metabolism in Mycobacterium tuberculosis. Front Mol Biosci 2:6. doi:10.3389/fmolb.2015.00006 25988174 PMC4428469

[29] Ruetz M, Campanello GC, Purchal M, Shen H, McDevitt L, Gouda H, Wakabayashi S, Zhu J, Rubin EJ, Warncke K, Mootha VK, Koutmos M, Banerjee R. 2019. Itaconyl-CoA forms a stable biradical in methylmalonyl-CoA mutase and derails its activity and repair. Science 366:589–593. doi:10.1126/science.aay0934 31672889 PMC7070230

[30] Priya M, Gupta SK, Koundal A, Kapoor S, Tiwari S, Kidwai S, Sorio de Carvalho LP, Thakur KG, Mahajan D, Sharma D, Kumar Y, Singh R. 2025. Itaconate mechanism of action and dissimilation in Mycobacterium tuberculosis Proc Natl Acad Sci USA 122:e2423114122. doi:10.1073/pnas.2423114122 39841148 PMC11789021

[31] Nair S, Huynh JP, Lampropoulou V, Loginicheva E, Esaulova E, Gounder AP, Boon AC, Schwarzkopf EA, Bradstreet TR, Edelson BT, Artyomov MN, Stallings CL, et al.. 2018. Irg1 expression in myeloid cells prevents immunopathology during M. tuberculosis infection. J Exp Med 215:1035–1045. doi:10.1084/jem.20180118 29511063 PMC5881474

[32] Michelucci A, Cordes T, Ghelfi J, Pailot A, Reiling N, Goldmann O, Binz T, Wegner A, Tallam A, Rausell A, Buttini M, Linster CL, Medina E, Balling R, Hiller K. 2013. Immune-responsive gene 1 protein links metabolism to immunity by catalyzing itaconic acid production. Proc Natl Acad Sci USA 110:7820–7825. doi:10.1073/pnas.1218599110 23610393 PMC3651434

[33] Shen H, Campanello GC, Flicker D, Grabarek Z, Hu J, Luo C, Banerjee R, Mootha VK. 2017. The human knockout gene CLYBL connects itaconate to vitamin B12. Cell 171:771–782. doi:10.1016/j.cell.2017.09.051 29056341 PMC5827971

[34] Mulholland CV, Wiggins TJ, Cui J, Vilchèze C, Rajagopalan S, Shultis MW, Reyes-Fernández EZ, Jacobs WR Jr, Berney M. 2024. Propionate prevents loss of the PDIM virulence lipid in Mycobacterium tuberculosis. Nat Microbiol 9:1607–1618. doi:10.1038/s41564-024-01697-8 38740932 PMC11253637

[35] Llibre A, Dedicoat M, Burel JG, Demangel C, O’Shea MK, Mauro C. 2021. Host immune-metabolic adaptations upon mycobacterial infections and associated co-morbidities. Front Immunol 12. doi:10.3389/fimmu.2021.747387 PMC8495197 34630426

[36] Bridwell-Rabb J, Drennan CL. 2017. Vitamin B 12 in the spotlight again. Curr Opin Chem Biol 37:63–70. doi:10.1016/j.cbpa.2017.01.013 28167430 PMC5540639

[37] Kipkorir T, Polgar P, Barker D, D’Halluin A, Patel Z, Arnvig KB. 2024. A novel regulatory interplay between atypical B12 riboswitches and uORF translation in Mycobacterium tuberculosis . Nucleic Acids Res 52:7876–7892. doi:10.1093/nar/gkae338 38709884 PMC11260477

[38] Kipkorir T, Mashabela GT, de Wet TJ, Koch A, Dawes SS, Wiesner L, Mizrahi V, Warner DF. 2021. De novo cobalamin biosynthesis, transport, and assimilation and cobalamin-mediated regulation of methionine biosynthesis in Mycobacterium smegmatis. J Bacteriol 203:e00620-20. doi:10.1128/JB.00620-20 33468593 PMC8088520

[39] Gopinath K, Venclovas Č, Ioerger TR, Sacchettini JC, McKinney JD, Mizrahi V, Warner DF. 2013. A vitamin B12 transporter in Mycobacterium tuberculosis. Open Biol 3. doi:10.1098/rsob.120175 PMC3603451 23407640

[40] Campos-Pardos E, Sanz-Asensio L, Pérez-Jiménez S, Yruela I, Contreras-Moreira B, Toledo-Arana A, Gonzalo-Asensio J. 2025. Evolutionary trajectories of methionine metabolism in Mycobacterium and its application to engineer a vitamin B12 whole-cell ribosensor. Microb Biotechnol 18:e70176. doi:10.1111/1751-7915.70176 40485089 PMC12146416

[41] Supply P, Marceau M, Mangenot S, Roche D, Rouanet C, Khanna V, Majlessi L, Criscuolo A, Tap J, Pawlik A, et al.. 2013. Genomic analysis of smooth tubercle bacilli provides insights into ancestry and pathoadaptation of Mycobacterium tuberculosis. Nat Genet 45:172–179. doi:10.1038/ng.2517 23291586 PMC3856870

[42] Brosch R, Gordon SV, Marmiesse M, Brodin P, Buchrieser C, Eiglmeier K, Garnier T, Gutierrez C, Hewinson G, Kremer K, Parsons LM, Pym AS, Samper S, van Soolingen D, Cole ST. 2002. A new evolutionary scenario for the Mycobacterium tuberculosis complex . Proc Natl Acad Sci USA 99:3684–3689. doi:10.1073/pnas.052548299 11891304 PMC122584

[43] Orgeur M, Brosch R. 2018. Evolution of virulence in the Mycobacterium tuberculosis complex. Curr Opin Microbiol 41:68–75. doi:10.1016/j.mib.2017.11.021 29216510

[44] Coscolla M, Gagneux S, Menardo F, Loiseau C, Ruiz-Rodriguez P, Borrell S, Otchere ID, Asante-Poku A, Asare P, Sánchez-Busó L, et al.. 2021. Phylogenomics of Mycobacterium africanum reveals a new lineage and a complex evolutionary history. Microb Genom 7:1–14. doi:10.1099/mgen.0.000477 PMC8208692 33555243

[45] Boritsch EC, Brosch R. 2016. Evolution of Mycobacterium tuberculosis: new insights into pathogenicity and drug resistance. Microbiol Spectr 4. doi:10.1128/microbiolspec.TBTB2-0020-2016 27787194

[46] Gagneux S. 2018. Ecology and evolution of Mycobacterium tuberculosis. Nat Rev Microbiol 16:202–213. doi:10.1038/nrmicro.2018.8 29456241

[47] Boritsch EC, Supply P, Honoré N, Seemann T, Stinear TP, Brosch R. 2014. A glimpse into the past and predictions for the future: the molecular evolution of the tuberculosis agent. Mol Microbiol 93:835–852. doi:10.1111/mmi.12720 25039682

[48] Izquierdo Lafuente B, Verboom T, Coenraads S, Ummels R, Bitter W, Speer A. 2024. Vitamin B_12_ uptake across the mycobacterial outer membrane is influenced by membrane permeability in Mycobacterium marinum Microbiol Spectr 12:e0316823. doi:10.1128/spectrum.03168-23 38722177 PMC11237697

[49] Ignatov DV, Salina EG, Fursov MV, Skvortsov TA, Azhikina TL, Kaprelyants AS. 2015. Dormant non-culturable Mycobacterium tuberculosis retains stable low-abundant mRNA. BMC Genomics 16:954. doi:10.1186/s12864-015-2197-6 26573524 PMC4647672

[50] Rodionov DA, Vitreschak AG, Mironov AA, Gelfand MS. 2003. Comparative genomics of the vitamin B12 metabolism and regulation in prokaryotes. Journal of Biological Chemistry 278:41148–41159. doi:10.1074/jbc.M305837200 12869542

[51] Watanabe F. 2007. Vitamin B12 sources and bioavailability. Exp Biol Med 232:1266–1274. doi:10.3181/0703-MR-67 17959839

[52] Finglas P. 1999. Bioavailability and analysis of vitamins in foods. Eur J Clin Nutr 53:80–81. doi:10.1038/sj.ejcn.1600596

[53] O’Sullivan JJ, Leeming RJ, Lynch SS, Pollock A. 1992. Radioimmunoassay that measures serum vitamin B12. J Clin Pathol 45:328–331. doi:10.1136/jcp.45.4.328 1577970 PMC495274

[54] Kolhouse JF, Kondo H, Allen NC, Podell E, Allen RH. 1978. Cobalamin analogues are present in human plasma and can mask cobalamin deficiency because current radioisotope dilution assays are not specific for true cobalamin. N Engl J Med 299:785–792. doi:10.1056/NEJM197810122991501 357970

[55] Hardlei TF, Nexo E. 2009. A new principle for measurement of cobalamin and corrinoids, used for studies of cobalamin analogs on serum haptocorrin. Clin Chem 55:1002–1010. doi:10.1373/clinchem.2008.114132 19299543

[56] Kolhouse JF, Allen RH. 1977. Absorption, plasma transport, and cellular retention of cobalamin analogues in the rabbit. Evidence for the existence of multiple mechanisms that prevent the absorption and tissue dissemination of naturally occurring cobalamin analogues. J Clin Invest 60:1381–1392. doi:10.1172/JCI108899 915005 PMC372496

[57] Kondo H, Kolhouse JF, Allen RH. 1980. Presence of cobalamin analogues in animal tissues (vitamin B-12/intrinsic factor/R protein/cobalt). Proc Natl Acad Sci USA 77:817–821. doi:10.1073/pnas.77.2.817 6928681 PMC348372

[58] Goig GA, Windels EM, Loiseau C, Stritt C, Biru L, Borrell S, Brites D, Gagneux S. 2025. Ecology, global diversity and evolutionary mechanisms in the Mycobacterium tuberculosis complex. Nat Rev Microbiol 23:602–614. doi:10.1038/s41579-025-01159-w 40133503

[59] Sweeney MI, Carranza CE, Tobin DM. 2025. Understanding Mycobacterium tuberculosis through its genomic diversity and evolution. PLoS Pathog 21:e1012956. doi:10.1371/journal.ppat.1012956 40019877 PMC11870338

[60] Pandey AK, Sassetti CM. 2008. Mycobacterial persistence requires the utilization of host cholesterol. Proc Natl Acad Sci USA 105:4376–4380. doi:10.1073/pnas.0711159105 18334639 PMC2393810

[61] Bomfim CCB, Fisher L, Amaral EP, Mittereder L, McCann K, Correa AAS, Namasivayam S, Swamydas M, Moayeri M, Weiss JM, Chari R, McVicar DW, Costa DL, D’Império Lima MR, Sher A. 2022. Mycobacterium tuberculosis induces Irg1 in murine macrophages by a pathway involving both TLR-2 and STING/IFNAR signaling and requiring bacterial phagocytosis. Front Cell Infect Microbiol 12:862582. doi:10.3389/fcimb.2022.862582 35586249 PMC9109611

[62] Xu L, Lin L, Xie N, Chen W, Nong W, Li R. 2024. Role of aryl hydrocarbon receptors in infection and inflammation. Front Immunol 15:1367734. doi:10.3389/fimmu.2024.1367734 38680494 PMC11045974

[63] Kim DJ, Venkataraman A, Jain PC, Wiesler EP, DeBlasio M, Klein J, Tu SS, Lee S, Medzhitov R, Iwasaki A. 2020. Vitamin B12 and folic acid alleviate symptoms of nutritional deficiency by antagonizing aryl hydrocarbon receptor. Proc Natl Acad Sci USA 117:15837–15845. doi:10.1073/pnas.2006949117 32571957 PMC7355044

[64] Eoh H, Rhee KY. 2014. Methylcitrate cycle defines the bactericidal essentiality of isocitrate lyase for survival of Mycobacterium tuberculosis on fatty acids. Proc Natl Acad Sci USA 111:4976–4981. doi:10.1073/pnas.1400390111 24639517 PMC3977286

[65] Muñoz-Elías EJ, McKinney JD. 2005. Mycobacterium tuberculosis isocitrate lyases 1 and 2 are jointly required for in vivo growth and virulence. Nat Med 11:638–644. doi:10.1038/nm1252 15895072 PMC1464426

[66] McKinney JD, Höner zu Bentrup K, Muñoz-Elías EJ, Miczak A, Chen B, Chan WT, Swenson D, Sacchettini JC, Jacobs WR Jr, Russell DG. 2000. Persistence of Mycobacterium tuberculosis in macrophages and mice requires the glyoxylate shunt enzyme isocitrate lyase. Nature 406:735–738. doi:10.1038/35021074 10963599

[67] Kwai BXC, Collins AJ, Middleditch MJ, Sperry J, Bashiri G, Leung IKH. 2021. Itaconate is a covalent inhibitor of the Mycobacterium tuberculosis isocitrate lyase. RSC Med Chem 12:57–61. doi:10.1039/d0md00301h 34046597 PMC8130629

[68] Wang H, Fedorov AA, Fedorov EV, Hunt DM, Rodgers A, Douglas HL, Garza-Garcia A, Bonanno JB, Almo SC, de Carvalho LPS. 2019. An essential bifunctional enzyme in Mycobacterium tuberculosis for itaconate dissimilation and leucine catabolism . Proc Natl Acad Sci USA 116:15907–15913. doi:10.1073/pnas.1906606116 31320588 PMC6689899

[69] Lin X, Lu D, Gao Y, Tao S, Yang X, Feng J, Tan A, Zhang H, Hu Y, Qin X, Kim S-T, Peng T, Mo L, et al.. 2012. Genome-wide association study identifies novel loci associated with serum level of vitamin B12 in Chinese men. Hum Mol Genet 21:2610–2617. doi:10.1093/hmg/dds062 22367966

[70] Lim ET, Würtz P, Havulinna AS, Palta P, Tukiainen T, Rehnström K, Esko T, Mägi R, Inouye M, Lappalainen T, et al.. 2014. Distribution and medical impact of loss-of-function variants in the Finnish founder population. PLoS Genet 10:e1004494. doi:10.1371/journal.pgen.1004494 25078778 PMC4117444

[71] Strittmatter L, Li Y, Nakatsuka NJ, Calvo SE, Grabarek Z, Mootha VK. 2014. CLYBL is a polymorphic human enzyme with malate synthase and β-methylmalate synthase activity. Hum Mol Genet 23:2313–2323. doi:10.1093/hmg/ddt624 24334609 PMC3976331

[72] Bradbeer C, Woodrow ML, Khalifah LI. 1976. Transport of vitamin B12 in Escherichia coli: common receptor system for vitamin B12 and bacteriophage BF23 on the outer membrane of the cell envelope. J Bacteriol 125:1032–1039. doi:10.1128/jb.125.3.1032-1039.1976 1254550 PMC236181

[73] de Veaux LC, Clevenson DS, Bradbeer C, Kadner RJ. 1986. Identification of the btuCED polypeptides and evidence for their role in vitamin B12 transport in Escherichia coli. J Bacteriol 167:920–927. doi:10.1128/jb.167.3.920-927.1986 3528128 PMC215959

[74] Cadieux N, Bradbeer C, Reeger-Schneider E, Köster W, Mohanty AK, Wiener MC, Kadner RJ. 2002. Identification of the periplasmic cobalamin-binding protein BtuF of Escherichia coli . J Bacteriol 184:706–717. doi:10.1128/JB.184.3.706-717.2002 11790740 PMC139523

[75] Noinaj N, Guillier M, Barnard, TJ, Buchanan SK. 2010. TonB-dependent transporters: regulation, structure, and function. Annu Rev Microbiol 64:43–60. doi:10.1146/annurev.micro.112408.134247 20420522 PMC3108441

[76] Rempel S, Stanek WK, Slotboom DJ. 2019. ECF-type ATP-binding cassette transporters. Annu Rev Biochem 88:551–576. doi:10.1146/annurev-biochem-013118-111705 30485755

[77] Slotboom DJ, Ettema TW, Nijland M, Thangaratnarajah C. 2020. Bacterial multi-solute transporters. FEBS Lett. Wiley Blackwell 594:3898–3907. doi:10.1002/1873-3468.13912 32810294

[78] Abellon-Ruiz J, Jana K, Silale A, Frey AM, Baslé A, Trost M, Kleinekathöfer U, van den Berg B. 2023. BtuB TonB-dependent transporters and BtuG surface lipoproteins form stable complexes for vitamin B_12_ uptake in gut Bacteroides. Nat Commun 14:4714. doi:10.1038/s41467-023-40427-2 37543597 PMC10404256

[79] Novichkov PS, Kazakov AE, Ravcheev DA, Leyn SA, Kovaleva GY, Sutormin RA, Kazanov MD, Riehl W, Arkin AP, Dubchak I, Rodionov DA. 2013. RegPrecise 3.0--a resource for genome-scale exploration of transcriptional regulation in bacteria. BMC Genomics 14:745. doi:10.1186/1471-2164-14-745 24175918 PMC3840689

[80] Rempel S, Gati C, Nijland M, Thangaratnarajah C, Karyolaimos A, de Gier JW, Guskov A, Slotboom DJ. 2020. A mycobacterial ABC transporter mediates the uptake of hydrophilic compounds. Nature 580:409–412. doi:10.1038/s41586-020-2072-8 32296172

[81] Nijland M, Lefebvre SN, Thangaratnarajah C, Slotboom DJ. 2024. Bidirectional ATP-driven transport of cobalamin by the mycobacterial ABC transporter BacA. Nat Commun 15:2626. doi:10.1038/s41467-024-46917-1 38521790 PMC10960864

[82] Domenech P, Kobayashi H, LeVier K, Walker GC, Barry CE 3rd. 2009. BacA, an ABC transporter involved in maintenance of chronic murine infections with Mycobacterium tuberculosis. J Bacteriol 191:477–485. doi:10.1128/JB.01132-08 18996991 PMC2620812

[83] Wuerges J, Garau G, Geremia S, Fedosov SN, Petersen TE, Randaccio L. 2006. Structural basis for mammalian vitamin B12 transport by transcobalamin. Proc Natl Acad Sci USA 103:4386–4391. doi:10.1073/pnas.0509099103 16537422 PMC1450181

[84] VanderVen BC, Huang L, Rohde KH, Russell DG. 2016. The minimal unit of infection: Mycobacterium tuberculosis in the macrophage. Microbiol Spectr 4. doi:10.1128/microbiolspec.TBTB2-0025-2016 PMC5245711 28084213

[85] Kipkorir T, Polgar P, D’Halluin A, Gap-Gaupool B, Makarov VA, Arnvig KB. 2024. The RpfB switch is a novel B12-sensing riboswitch regulating (non-replicating) persistence in Mycobacterium tuberculosis. bioRxiv. doi:10.1101/2024.07.19.603033

[86] Quadros EV. 2010. Advances in the understanding of cobalamin assimilation and metabolism. Br J Haematol 148:195–204. doi:10.1111/j.1365-2141.2009.07937.x 19832808 PMC2809139

[87] Zheng W, Chang IC, Limberis J, Budzik JM, Zha BS, Howard Z, Chen L, Ernst JD. 2024. Mycobacterium tuberculosis resides in lysosome-poor monocyte-derived lung cells during chronic infection. PLoS Pathog 20:e1012205. doi:10.1371/journal.ppat.1012205 38701094 PMC11095722

[88] Wexler AG, Schofield WB, Degnan PH, Folta-Stogniew E, Barry NA, Goodman AL. 2018. Human gut bacteroides capture vitamin B12 via cell surface-exposed lipoproteins. eLife 7:e37138. doi:10.7554/eLife.37138 30226189 PMC6143338

[89] Mascarenhas R, Ruetz M, McDevitt L, Koutmos M, Banerjee R. 2020. Mobile loop dynamics in adenosyltransferase control binding and reactivity of coenzyme B_12_ Proc Natl Acad Sci USA 117:30412–30422. doi:10.1073/pnas.2007332117 33199623 PMC7720225

[90] Banerjee R, Gouda H, Pillay S. 2021. Redox-linked coordination chemistry directs vitamin B_12_ trafficking. Acc Chem Res 54:2003–2013. doi:10.1021/acs.accounts.1c00083 33797888 PMC8142554

[91] Koutmos M, Gherasim C, Smith JL, Banerjee R. 2011. Structural basis of multifunctionality in a vitamin B12-processing enzyme. J Biol Chem 286:29780–29787. doi:10.1074/jbc.M111.261370 21697092 PMC3191019

[92] Balabanova L, Averianova L, Marchenok M, Son O, Tekutyeva L. 2021. Microbial and genetic resources for cobalamin (vitamin B12) biosynthesis: from ecosystems to industrial biotechnology. Int J Mol Sci 22:4522. doi:10.3390/ijms22094522 33926061 PMC8123684

[93] Rempel S, Colucci E, de Gier JW, Guskov A, Slotboom DJ. 2018. Cysteine-mediated decyanation of vitamin B12 by the predicted membrane transporter BtuM. Nat Commun 9. doi:10.1038/s41467-018-05441-9 PMC6072759 30072686

[94] Martínez Felices JM, Barreto YB, Thangaratnarajah C, Whittaker JJ, Alencar AM, Guskov A, Slotboom DJ. 2024. Cobalamin decyanation by the membrane transporter BtuM. Structure 32:1165–1173. doi:10.1016/j.str.2024.04.014 38733996

[95] Kim J, Gherasim C, Banerjee R. 2008. Decyanation of vitamin B12 by a trafficking chaperone. Proceedings of the National Academy of Sciences 105:14551–14554. doi:10.1073/pnas.0805989105 PMC2567227 18779575

[96] Campbell GRO, Taga ME, Mistry K, Lloret J, Anderson PJ, Roth JR, Walker GC. 2006. Sinorhizobium meliloti bluB is necessary for production of 5,6-dimethylbenzimidazole, the lower ligand of B12. Proc Natl Acad Sci USA 103:4634–4639. doi:10.1073/pnas.0509384103 16537439 PMC1450223

[97] Taga ME, Larsen NA, Howard-Jones AR, Walsh CT, Walker GC. 2007. BluB cannibalizes flavin to form the lower ligand of vitamin B12. Nature 446:449–453. doi:10.1038/nature05611 17377583 PMC2770582

[98] Dudko D, Milker S, Holtmann D, Buchhaupt M. 2023. Identification of vitamin B_12_ producing bacteria based on the presence of bluB/cobT2 homologues. Biotechnol Lett 45:563–572. doi:10.1007/s10529-023-03362-2 36913101 PMC10038948

[99] Szklarczyk D, Kirsch R, Koutrouli M, Nastou K, Mehryary F, Hachilif R, Gable AL, Fang T, Doncheva NT, Pyysalo S, Bork P, Jensen LJ, von Mering C. 2023. The STRING database in 2023: protein-protein association networks and functional enrichment analyses for any sequenced genome of interest. Nucleic Acids Res 51:D638–D646. doi:10.1093/nar/gkac1000 36370105 PMC9825434

[100] Tsang AW, Escalante-Semerena JC. 1999. CobB, a new member of the SIR2 family of eucaryotic regulatory proteins, is required to compensate for the lack of nicotinate mononucleotide:5,6-dimethylbenzimidazole phosphoribosyltransferase activity in cobT mutants during cobalamin biosynthesis in Salmonella typhimurium LT2. J Biol Chem 273:31788–31794. doi:10.1074/jbc.273.48.31788 9822644

[101] Trzebiatowski JR, O’Toole GA, Escalante-Semerena JC. 1994. The cobT gene of Salmonella typhimurium encodes the NaMN: 5,6-dimethylbenzimidazole phosphoribosyltransferase responsible for the synthesis of N1-(5-phospho-alpha-D-ribosyl)-5,6-dimethylbenzimidazole, an intermediate in the synthesis of the nucleotide loop of cobalamin. J Bacteriol 176:3568–3575. doi:10.1128/jb.176.12.3568-3575.1994 8206834 PMC205545

[102] Maggio-Hall LA, Escalante-Semerena JC. 2003. Alpha-5,6-dimethylbenzimidazole adenine dinucleotide (alpha-DAD), a putative new intermediate of coenzyme B12 biosynthesis in Salmonella typhimurium. Microbiology (Reading) 149:983–990. doi:10.1099/mic.0.26040-0 12686640

[103] Pawełczyk J, Brzostek A, Minias A, Płociński P, Rumijowska-Galewicz A, Strapagiel D, Zakrzewska-Czerwińska J, Dziadek J. 2021. Cholesterol-dependent transcriptome remodeling reveals new insight into the contribution of cholesterol to Mycobacterium tuberculosis pathogenesis. Sci Rep 11:12396. doi:10.1038/s41598-021-91812-0 34117327 PMC8196197

[104] Nahvi A, Barrick JE, Breaker RR. 2004. Coenzyme B12 riboswitches are widespread genetic control elements in prokaryotes. Nucleic Acids Res 32:143–150. doi:10.1093/nar/gkh167 14704351 PMC373277

[105] Johnson JE, Reyes FE, Polaski JT, Batey RT. 2012. B12 cofactors directly stabilize an mRNA regulatory switch. Nature 492:133–137. doi:10.1038/nature11607 23064232 PMC3518761

[106] McCown PJ, Corbino KA, Stav S, Sherlock ME, Breaker RR. 2017. Riboswitch diversity and distribution. RNA 23:995–1011. doi:10.1261/rna.061234.117 28396576 PMC5473149

[107] Polaski JT, Webster SM, Johnson JE, Batey RT. 2017. Cobalamin riboswitches exhibit a broad range of ability to discriminate between methylcobalamin and adenosylcobalamin. J Biol Chem 292:11650–11658. doi:10.1074/jbc.M117.787176 28483920 PMC5512062

[108] Bhat KH, Das A, Srikantam A, Mukhopadhyay S. 2013. PPE2 protein of Mycobacterium tuberculosis may inhibit nitric oxide in activated macrophages. Ann N Y Acad Sci 1283:97–101. doi:10.1111/nyas.12070 23448669

[109] Bhat KH, Srivastava S, Kotturu SK, Ghosh S, Mukhopadhyay S. 2017. The PPE2 protein of Mycobacterium tuberculosis translocates to host nucleus and inhibits nitric oxide production. Sci Rep 7. doi:10.1038/srep39706 PMC5223167 28071726

[110] Srivastava S, Battu MB, Khan MZ, Nandicoori VK, Mukhopadhyay S. 2019. Mycobacterium tuberculosis PPE2 protein interacts with p67phox and inhibits reactive oxygen species production . The Journal of Immunology 203:1218–1229. doi:10.4049/jimmunol.1801143 31375544

[111] Pal R, Mukhopadhyay S. 2021. PPE2 protein of Mycobacterium tuberculosis affects myeloid hematopoiesis in mice. Immunobiology 226:152051. doi:10.1016/j.imbio.2020.152051 33352401

[112] Schwenk S, Moores A, Nobeli I, McHugh TD, Arnvig KB. 2018. Cell-wall synthesis and ribosome maturation are co-regulated by an RNA switch in Mycobacterium tuberculosis. Nucleic Acids Res 46:5837–5849. doi:10.1093/nar/gky226 29618088 PMC6009663

[113] Cotta KB, Ghosh S, Mehra S. 2022. Potentiating the anti-tuberculosis efficacy of peptide nucleic acids through combinations with permeabilizing drugs. Microbiol Spectr 10:e0126221. doi:10.1128/spectrum.01262-21 35171048 PMC8849056

[114] Shell TA, Lawrence DS. 2015. Vitamin B12: a tunable, long wavelength, light-responsive platform for launching therapeutic agents. Acc Chem Res 48:2866–2874. doi:10.1021/acs.accounts.5b00331 26479305 PMC5240631

[115] Pieńko T, Czarnecki J, Równicki M, Wojciechowska M, Wierzba AJ, Gryko D, Bartosik D, Trylska J. 2021. Vitamin B_12_-peptide nucleic acids use the BtuB receptor to pass through the Escherichia coli outer membrane. Biophys J 120:725–737. doi:10.1016/j.bpj.2021.01.004 33453274 PMC7896106

[116] Równicki M, Dabrowska Z, Wojciechowska M, Wierzba AJ, Maximova K, Gryko D, Trylska J. 2019. Inhibition of Escherichia coli growth by vitamin B12-peptide nucleic acid conjugates. ACS Omega 4:819–824. doi:10.1021/acsomega.8b03139

[117] Równicki M, Wojciechowska M, Wierzba AJ, Czarnecki J, Bartosik D, Gryko D, Trylska J. 2017. Vitamin B_12_ as a carrier of peptide nucleic acid (PNA) into bacterial cells. Sci Rep 7:7644. doi:10.1038/s41598-017-08032-8 28794451 PMC5550456

[118] Clardy SM, Allis DG, Fairchild TJ, Doyle RP. 2011. Vitamin B12 in drug delivery: breaking through the barriers to a B12 bioconjugate pharmaceutical. Expert Opin Drug Deliv 8:127–140. doi:10.1517/17425247.2011.539200 21128823

[119] Guzzo MB, Nguyen HT, Pham TH, Wyszczelska-Rokiel M, Jakubowski H, Wolff KA, Ogwang S, Timpona JL, Gogula S, Jacobs MR, Ruetz M, Kräutler B, Jacobsen DW, Zhang GF, Nguyen L. 2016. Methylfolate trap promotes bacterial thymineless death by sulfa drugs. PLoS Pathog 12:e1005949. doi:10.1371/journal.ppat.1005949 27760199 PMC5070874

[120] Boot M, Sparrius M, Jim KK, Commandeur S, Speer A, van de Weerd R, Bitter W. 2016. iniBAC induction Is vitamin B12- and MutAB-dependent in Mycobacterium marinum. Journal of Biological Chemistry 291:19800–19812. doi:10.1074/jbc.M116.724088 27474746 PMC5025670

[121] Degnan PH, Barry NA, Mok KC, Taga ME, Goodman AL. 2014. Human gut microbes use multiple transporters to distinguish vitamin B₁₂ analogs and compete in the gut. Cell Host Microbe 15:47–57. doi:10.1016/j.chom.2013.12.007 24439897 PMC3923405

[122] Deery E, Lawrence AD, Warren MJ. 2022. Biosynthesis of cobamides: methods for the detection, analysis and production of cobamides and biosynthetic intermediates, p 3–23. In Methods in enzymology. Academic Press Inc. 10.1016/bs.mie.2022.01.013 35589198

[123] Sobczyńska-Malefora A, Delvin E, McCaddon A, Ahmadi KR, Harrington DJ. 2021. Vitamin B_12_ status in health and disease: a critical review. Diagnosis of deficiency and insufficiency - clinical and laboratory pitfalls. Crit Rev Clin Lab Sci 58:399–429. doi:10.1080/10408363.2021.1885339 33881359

[124] Lawrence AD, Nemoto-Smith E, Deery E, Baker JA, Schroeder S, Brown DG, Tullet JMA, Howard MJ, Brown IR, Smith AG, Boshoff HI, Barry CE, Warren MJ. 2018. Construction of fluorescent analogs to follow the uptake and distribution of cobalamin (vitamin B_12_) in bacteria, worms, and plants. Cell Chem Biol 25:941–951. doi:10.1016/j.chembiol.2018.04.012 29779954 PMC6125784

[125] Quispe Haro JJ, Wegner SV. 2023. An adenosylcobalamin specific whole-cell biosensor. Adv Healthc Mater 12:e2300835. doi:10.1002/adhm.202300835 37070155 PMC11468855

